# Fungal acetylome comparative analysis identifies an essential role of acetylation in human fungal pathogen virulence

**DOI:** 10.1038/s42003-019-0419-1

**Published:** 2019-05-01

**Authors:** Yanjian Li, Hailong Li, Mingfei Sui, Minghui Li, Jiamei Wang, Yang Meng, Tianshu Sun, Qiaojing Liang, Chenhao Suo, Xindi Gao, Chao Li, Zhuoran Li, Wei Du, Baihua Zhang, Sixiang Sai, Zhang Zhang, Jing Ye, Hongchen Wang, Shang Yue, Jiayi Li, Manli Zhong, Changbin Chen, Shouliang Qi, Ling Lu, Dancheng Li, Chen Ding

**Affiliations:** 1 College of Life and Health Sciences, Northeastern University, Shenyang, China; 2 Software College, Northeastern University, Shenyang, China; 3 Department of Scientific Research, Central Laboratory, Peking Union Medical College Hospital, Chinese Academy of Medical Science, Beijing, China; 4 Beijing Key Laboratory for Mechanisms Research and Precision Diagnosis of Invasive Fungal Diseases, Beijing, China; 5 SINO-Dutch Biomedical and Information Engineering School, Northeastern University, Shenyang, China; 6 School of Medicine, Binzhou Medical University, Yantai, China; 7 Jingjie PTM Biolab Co. Ltd, Hangzhou, China; 8 Jiangsu Key Laboratory for Microbes and Functional Genomics, Jiangsu Engineering and Technology Research Center for Microbiology, College of Life Sciences, Nanjing Normal University, Nanjing, China; 9 Neural Plasticity and Repair Unit, Wallenberg Neuroscience Center, Lund University, Lund, Sweden; 10 Unit of Pathogenic Fungal Infection & Host Immunity, CAS Key Laboratory of Molecular Virology & Immunology, Institute Pasteur of Shanghai, Chinese Academy of Science, Shanghai, China; 11 Key Laboratory of Data Analytics and Optimization for Smart Industry, Northeastern University, Shenyang, China

**Keywords:** Fungal biology, Coevolution, Acetyltransferases

## Abstract

Lysine acetylation is critical in regulating important biological processes in many organisms, yet little is known about acetylome evolution and its contribution to phenotypic diversity. Here, we compare the acetylomes of baker’s yeast and the three deadliest human fungal pathogens, *Cryptococcus neoformans*, *Candida albicans*, and *Aspergillus fumigatus*. Using mass spectrometry enriched for acetylated peptides together with public data from *Saccharomyces cerevisiae*, we show that fungal acetylomes are characterized by dramatic evolutionary dynamics and limited conservation in core biological processes. Notably, the levels of protein acetylation in pathogenic fungi correlate with their pathogenicity. Using gene knockouts and pathogenicity assays in mice, we identify deacetylases with critical roles in virulence and protein translation elongation. Finally, through mutational analysis of deactylation motifs we find evidence of positive selection at specific acetylation motifs in fungal pathogens. These results shed new light on the pathogenicity regulation mechanisms underlying the evolution of fungal acetylomes.

## Abstract (web summary)

Yanjian Li and Hailong Li, et al. report interspecific comparisons of the acetylomes of yeast and three human fungal pathogens. Through evolutionary analysis and experimental validation, they show that pathogen-specific aceylation motifs and deacetylase genes are required for normal virulence in an animal infection model.

## Introduction

Protein lysine acetylation (Kac) is a reversible posttranslational protein modification that is critical for many biological processes and cell activities that are linked to important human diseases, including histone modification, glycolysis, ferroptosis, tumor differentiation, infectious diseases, and others^1–4^. Despite the discovery of protein acetylation 50 years ago, little is known about the evolution of the acetylome in nature, and the contribution of acetylome evolution to phenotypic diversity of a broad number of species remains uninvestigated. How do acetylomes coevolve with organism behaviors, and what is the contribution of acetylome evolution to an organism’s adaptation to its environment? Lack of both a protein Kac database and an interspecies comparative Kac analysis in major model organisms are significant obstacles when trying to answer these questions.

Fungi are invaluable models for understanding evolution and its role in modulating cell adaptation to different environments. Critical genome-based evolutionary discoveries have been reported using fungal cells^5–7^. In the fungal kingdom, *Saccharomyces cerevisiae*, *Cryptococcus neoformans*, *Aspergillus fumigatus*, and *Candida albicans* are major model species. Notably, *Cryptococcus*, *Aspergillus*, and *Candida* spp. are the deadliest infectious fungal pathogens for humans, serving as clinically important and useful research models for studying fungal pathogenesis^5,8,9^. The airborne human pathogens, *C. neoformans* and *A. fumigatus*, are widely distributed worldwide and are causative agents of severe lung infections^9,10^. *C. neoformans* can cause lethal meningitis in both immunocompetent and immunodeficient individuals^11,12^. *C. albicans* is recognized as a commensal fungus in a majority of the human population, but it can become a causative agent of bloodstream, skin, oral and gastrointestinal infections^13,14^. These fungal species span a dramatic and wide evolutionary range and demonstrate distinct phenotypic differences such as cell morphological changes, pathogenicity, and cell biology, and they can provide valuable tools for analyzing acetylome evolutionary mechanisms.

Accumulating evidence has indicated the importance of acetylation in fungal biology. In *C. albicans*, acetylation deacetylase, Hda1, is involved in phenotypic plasticity via the target of rapamycin signaling pathway. Genetic and pharmacological alterations in *C. albicans* H3K56ac affect fungal proliferation and the yeast’s fitness in the host^15,16^. *C. albicans* Sir2 mediates genome stability at the subtelomere region^17^. *A*. *fumigatus* HdaA acts as an important modulator in germination and secondary metabolism^18^. *A. fumigatus* heat shock protein Hsp90 acetylation participates in modulation of drug resistance^19^. Deacetylase inhibitors are capable of inhibiting the production of virulence factors in *C. neoformans*^20^. These new results may shed new light on acetylation as a potential therapeutic target for anti-fungal drug development^21,22^. However, systematic understanding of the fungal acetylome is hampered by several major deficiencies. Given that these divergent related fungal species have undergone dynamic evolutionary changes, how did acetylomes evolve in fungi and do fungi share evolutionary conservation in acetylomes? How do distinct fungal life cycles coevolve with their acetylomes? What is the major effect of deacetylase inhibitors as anti-fungal agents, and do they work through inhibition of virulence producing machinery or certain core biological fungal processes, and most importantly, do the inhibitory effects block conserved acetylated processes in the host cells?

In order to systematically understand fungal acetylomes and their evolution, we performed a comprehensive interspecies comparison of acetylomes using four major fungal model organisms. Our data demonstrated that fungal acetylomes exhibit dynamic evolutionary phenomenon. We analyzed two fungal conservation levels: orthologous (Kac) conservation and Kac site conservation. Together, 350 fungal orthologs possess Kac modifications, whereas conserved Kac sites are limited to only 126 sites. These results suggest that fungal acetylomes are highly dynamic events. Although the Kac modifications of the orthologs that are involved in core biological processes, including protein translation, histone modifications, tricarboxylic acid (TCA) cycle, and respiration are conserved across species, the Kac sites are only partially conserved. Intriguingly, despite the highly dynamic occurrence of fungal acetylation sites, *C. neoformans*, *C. albicans*, and *A. fumigatus* reveal that the acetylomes of pathogenic fungi share commonly conserved Kac motifs that are different from those in *S. cerevisiae*. Interestingly, pathogen-specific Kac motifs coincide with the incidence of identified fungal virulence factors, which indicate a novel form of selective pressure during pathogenic fungi evolution. Consequently, mutating pathogen-specific Kac motifs in *C. neoformans* result in the attenuation of the fitness of the mutants in the host.

## Results

### Interspecies comparative acetylomes in four fungal species

**Fig. 1:**
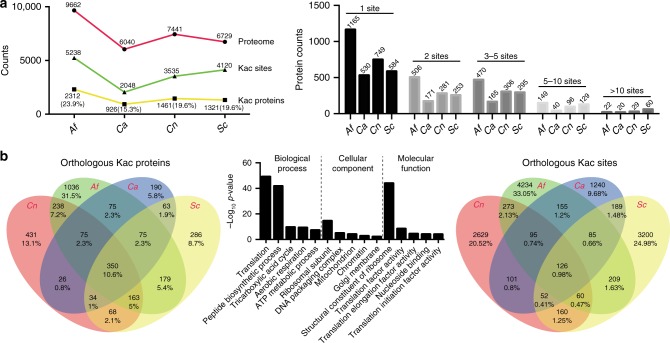
Interspecies comparative acetylome analysis. **a** Survey of the acetylomes and Kac sites of the four fungal species. **b** Comparison of Kac orthologs and sites among the four fungal species. The conserved orthologous Kac proteins were identified based on acetylome data and interspecies orthologous relationships. A gene ontology (GO) analysis was performed using 350 conserved orthologs. Representative GO terms of biological process, cellular components, and molecular function were plotted. The conserved orthologous Kac sites were defined as conserved acetylated lysine residues, which were identified based on the acetylome data and multiple orthologous alignments

To decipher fungal acetylome evolution, we used protein samples from *C. neoformans*, *C. albicans*, and *A. fumigatus* model organisms, representing distantly related fungal species. In the analysis of the acetylome in *C. albicans*, the protein samples isolated from both hyphal and yeast cells were pooled, allowing the global detection of acetylated proteins. We applied a Kac pan antibody for the enrichment of acetylated peptides followed by mass spectrometry for Kac protein detection. An interspecies comparative acetylome analysis was performed in which data from three major fungal pathogens and the published data of baker’s yeast^23,24^ were used (Supplementary Fig. 1, Supplementary Data 1). While the proteome sizes of *C. albicans*, *S. cerevisiae*, and *C. neoformans* are comparable, *C. albicans* has the fewest Kac sites and acetylated proteins (Fig. 1a). The *A. fumigatus* proteome contains the most Kac sites among all of the tested fungi, which is likely due to its large proteome. We analyzed the orthologs of each acetylated protein in all of the acetylome data, and the comparison revealed that Kac sites from 350 orthologs (10.6% of total detected orthologs) were detected in all 4 of the studied fungi, and only 75 orthologs were shared by the pathogenic fungi (2.3% of total orthologs) (Fig. 1b, Supplementary Data 2). The highly conserved 350 Kac orthologs were enriched in core biological processes, such as translation, TCA, respiration, and ribosome and histone modifications (Fig. 1b). Of the 350 conserved Kac proteins, the K resides of only 126 Kac sites (0.98% of total Kac sites) were conserved in the orthologs of core fungal biological processes (Fig. 1b, Supplementary Data 2). Also, each fungal species possessed species-specific acetylated orthologs (13.1% for *C. neoformans*, 31.5% for *A. fumigatus*, 5.8% for *C. albicans*, and 8.7% for *S. cerevisiae*) and unique Kac sites (2629 Kac unique sites for *C. neoformans*, 1240 sites for *C. albicans*, 4234 sites for *A. fumigatus*, and 3200 sites for *S. cerevisiae*) (Fig. 1b). Our analysis suggests that fungal Kac sites span a wide evolutionary range and demonstrate large differences among fungi.

### Fungal translation elongation processes are regulated by Kac

**Fig. 2:**
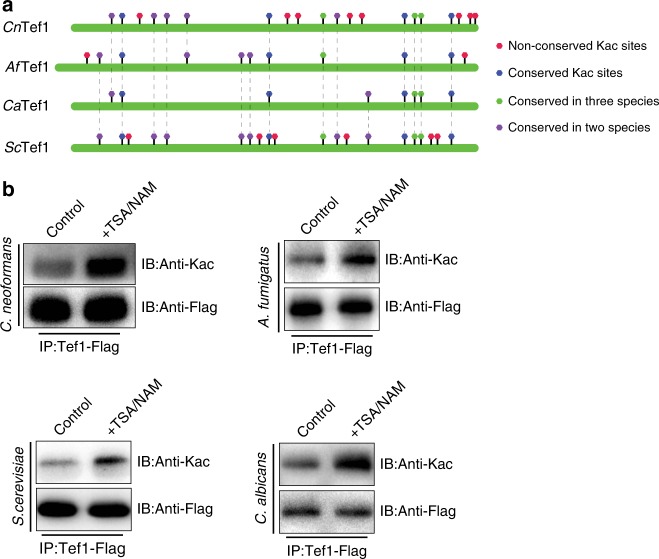
Kac is conserved in fungal Tef1 orthologs. **a** Map of Tef1 Kac sites. Conservation of Tef1 Kac sites is indicated. **b** Confirmation of Kac in Tef1 orthologs. Tef1 proteins from *TEF1-FLAG* strains of *S. cerevisiae*, *C. neoformans*, *C. albicans*, and *A. fumigatus* were pulled down and quantified. Full blots are shown in Supplementary Fig. 2c

The data for fungal acetylome showed that proteins involved in essential or core biological processes contain conserved Kac sites. Protein translation is the most highly enriched process in the gene ontology analysis. To verify the conservation of Kac orthologs, a critical protein for protein translation (Tef1) was selected for further characterization because Tef1 orthologs possess the most conserved Kac sites among the orthologs chosen for evaluation (Supplementary Data 2). Tef1 is a highly conserved fungal protein, and our acetylome comparison analysis revealed that elongation factors are hyperacetylated in fungi with interspecies Kac site variation (Fig. 2a and Supplementary Fig. 2a, Supplementary Data 1 and 2). In order to address Kac properties of Tef1 orthologs, *TEF1-FLAG* strains were constructed for *S. cerevisiae*, *C. neoformans*, *C. albicans*, and *A. fumigatus* (Supplementary Fig. 2b). Protein pull-down assays revealed that Kac levels are induced when exposing cells to deacetylase inhibitors, demonstrating that Tef1 Kac sites are an evolutionarily conserved posttranslation modification in these fungi (Fig. 2b).

**Fig. 3:**
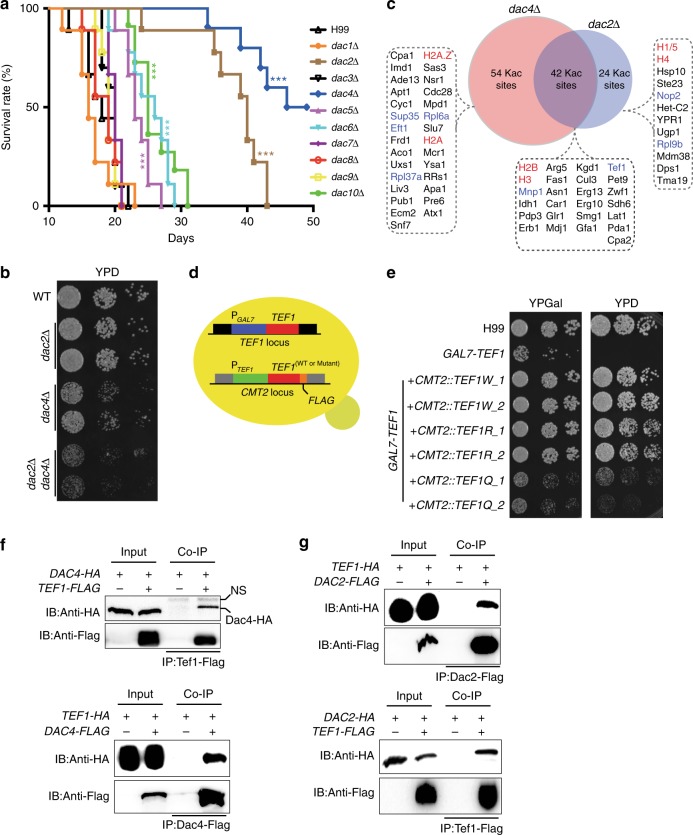
*C. neoformans* deacetylases regulate Tef1 function. **a** Animal survival assays. Mice (*n* = 10) were infected with *C. neoformans* deacetylase mutants. Mice survival rates were recorded. A Kaplan–Meier survival chart was plotted. **b** Spotting assay of *DAC2* and *DAC4* mutants. Strains were spotted onto yeast glucose (YPD) agar. The photograph was taken after two days of incubation. **c** The comparative acetylome analysis of Dac2 and Dac4. Identified proteins were indicated, in which red shows histones and blue indicates proteins involved in protein synthesis. **d** Generation of *C. neoformans TEF1-FLAG* strain. The native promoter of *TEF1* was replaced with a *GAL7* promoter. The resulting strain was transformed with a wild-type *TEF1* or a Q or R mutation gene with the native promoter and a C-terminal Flag DNA sequence at the *CMT2* locus. **e** Spotting assays of *TEF1* mutants. Strains were spotted onto yeast galactose (YPGal) and YPD agars. The plates were incubated at 30 °C for 2 days. **f** Co-immunoprecipitation (Co-IP) of Dac4 and Tef1. Co-IP assays were performed in the strains *DAC4-HA/TEF1-FLAG* and *DAC4-FLAG/TEF1-HA*. Proteins were pulled down using Flag beads. Anti-HA and anti-Flag antibodies were used. NS indicates nonspecific bands. Full blots are shown in Supplementary Fig. 3e. **g** Co-IP of Dac2 and Tef1. The assay was performed as described in Fig. 3f, except that the strains *DAC2-HA/TEF1-FLAG* and *DAC2-FLAG/TEF1-HA* were used. Full blots are shown in Supplementary Fig. 3e

We therefore reasoned that deacetylases are critical regulators in the modulation of protein synthetic processes. To address this hypothesis, we used *C. neoformans* genetic tools in order to generate deacetylase knockout mutants. Because Tef1 is an essential protein, we hypothesized that disturbing Tef1 acetylation levels would result in impairment of Tef1 protein activity, which in turn, would give rise to fungal pathogenicity defects. Therefore, *C. neoformans* deacetylase knockout mutants were screened for fungal pathogenicity in mice, demonstrating that *DAC2* and *DAC4* are critical regulators of fungal fitness in the host (Fig. 3a; discussed below). Furthermore, the *dac2Δ dac4Δ* strain showed significant cell growth inhibition, implicating that Tef1 is a client protein for division arrest, controlled by Dac2 and Dac4 in *C. neoformans* (Fig. 3b).

Acetylome quantification analyses were performed in order to determine the regulatory mechanisms of Tef1 via Dac2 and Dac4 (Supplementary Data 1, Supplementary Fig. 3a, b). In the acetylome data, the levels of acetylated peptides were quantified and normalized to the proteome data. The acetylation of 96 K sites on 69 proteins was increased in *dac4Δ* while 66 K sites from 46 proteins demonstrated increased acetylation in *dac2Δ*. The acetylome data of *dac2Δ* and *dac4Δ* revealed that 42 Kac sites from 31 proteins were co-regulated by Dac2 and Dac4. Dac2 and Dac4 regulate a list of histones and proteins involved in protein synthesis (such as Tef1, mitochondrial nucleoid protein Mnp1, eukaryotic translation release factor Sup35) (Fig. 3c, Supplementary Data 1).

Kac acetylation in Tef1 was induced in both *dac2Δ* and *dac4Δ* strains (Fig. 3c and Supplementary Fig. 3b), which suggest a novel form of regulatory machinery in protein synthesis. Three Kac sites from Tef1’s GTP-binding domain (K36, K41, and K217) were upregulated (by more than two-fold) in *dac2Δ* cells, and Dac4 appears to control K41 and K217 sites (Supplementary Fig. 3b). In order to explore the function of these Kac sites further, we constructed Tef1 mutants (Fig. 3d). Because Tef1 is an essential gene, we first replaced the endogenous *TEF1* promoter with the *GAL7* promoter, which generated the *GAL7-TEF1* strain. Second, the wild-type *TEF1* gene tagged with a *FLAG* was cloned and integrated at the *CMT2* locus, yielding the *GAL7-TEF1/TEF1W* strain in which the *GAL7*-*TEF1* is repressed by glucose, which allows solo expression of the Tef1-Flag from the *CMT2* locus (Fig. 3d). To address the function of Kac in Tef1, we integrated Tef1 mutant genes (K36, K41, and K217 to Q or R) into the *GAL7-TEF1* strain, generating *GAL7-TEF1/TEF1Q* (K-to-Q mutation) and *GAL7-TEF1/TEF1R* (K-to-R mutation) strains (Fig. 3d). We demonstrated that the *GAL7-TEF1* strain transformed with *TEF1W* or *TEF1R* could fully rescue cell growth on both yeast peptone galactose (YPGal) and yeast peptone glucose (YPD) media. However, the *TEF1Q* mutant was unable to restore wild-type Tef1 cell growth even when robust protein expression was detected (Fig. 3e and Supplementary Fig. 3c) and resembled that of the *dac2Δ dac4Δ* mutant. This result suggests that acetylation at K36, K41, and K217 is essential for Tef1 function in *C. neoformans*.

Next, we performed protein co-immunoprecipitation assays in order to determine whether the regulation of Tef1 Kac by Dac2 and Dac4 occurs via direct interactions. Our results reveal that Dac2 and Dac4 directly interact with *C. neoformans* Tef1 (Supplementary Fig. 3d, e and Fig. 3f, g), which indicates that Dac2 and Dac4 are important for Tef1 function. Consistent with these analyses, the Kac levels of Tef1 is dramatically induced when disrupting *DAC2* or *DAC4* (Supplementary Fig. 3f). These results demonstrate that deacetylases have essential functions in protein synthesis processes via the modification of elongation factors in fungal species.

### Kac is a critical regulator of fungal pathogenicity

**Fig. 4:**
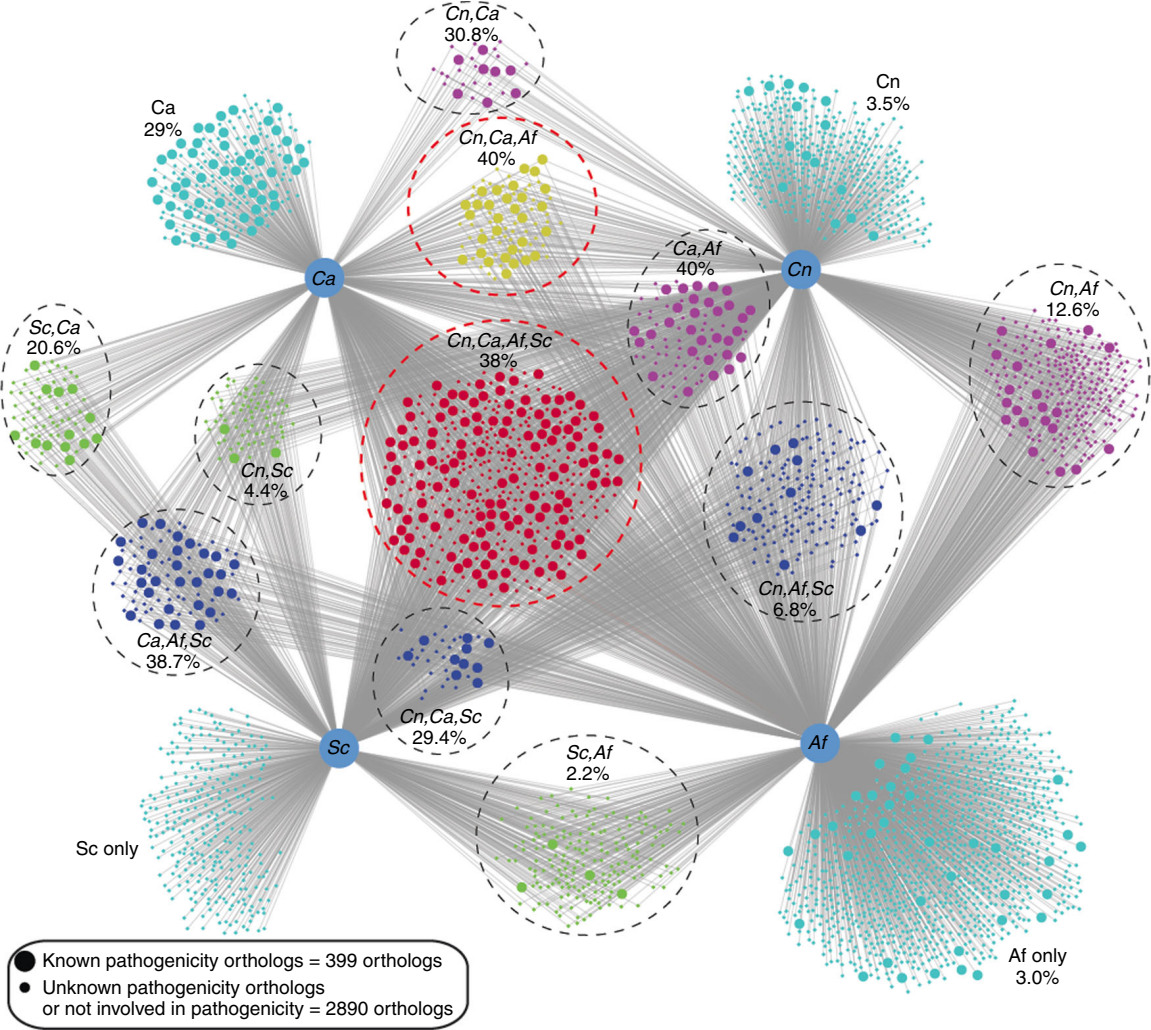
Fungal pathogenicity factor identified in acetylomes. Comparative acetylome networks in four fungal species. The orthologous relationship of the acetylated proteins of four fungal species was downloaded from FungiDB (http://fungidb.org/fungidb/)^45^ and OrthoMCL (http://orthomcl.org/orthomcl/)^46^. The network was constructed using Cytoscape with literature information integrated for each ortholog protein. Large dots represent pathogenicity-associated phenotypes for at least one ortholog from one organism. Small dots represent either no function or unknown function in fungal pathogenicity. The percentage of pathogenicity factors in each group is indicated

*C. neoformans*, *A. fumigatus*, and *C. albicans* are major human fungal pathogens. Fungal fitness is defined as the ability to colonize and proliferate in and invade the host during systemic infection. Fungal fitness is a major aspect of fungal life cycle and biology. In order to globally decipher the correlation between Kac and fungal pathogenicity, the function of each acetylated ortholog in fungal pathogenicity was examined and integrated to generate a pathogenicity–acetylome network (Fig. 4, Supplementary Data 1, 2, and 3). This analysis revealed that the reported pathogenicity-related orthologs are primarily concentrated in the group of acetylated proteins conserved in all species and in the group shared by the pathogenic fungi, which had 38% and 40% of pathogenicity factors, respectively (Fig. 4, Supplementary Data 3). Collectively, 399 orthologs have been identified as pathogenicity modulators in at least one human fungal pathogen, and their functions during the onset of infectious disease is potentially balanced by Kac, implicating Kac as an important regulator of fungal fitness during infection.

In order to extensively investigate the molecular mechanisms of Kac regulation that are involved in fungal pathogenicity, we assessed biological traits in *C. neoformans* after treatment with two specific deacetylase inhibitors, trichostatin A (TSA) and nicotinamide (NAM). We tested for the presence of melanin and capsule alterations in response to TSA/NAM. While melanin was completely abolished (Supplementary Fig. 4a), capsule thickness was reduced by approximately 30% (Supplementary Figs. 4b, c).

**Fig. 5:**
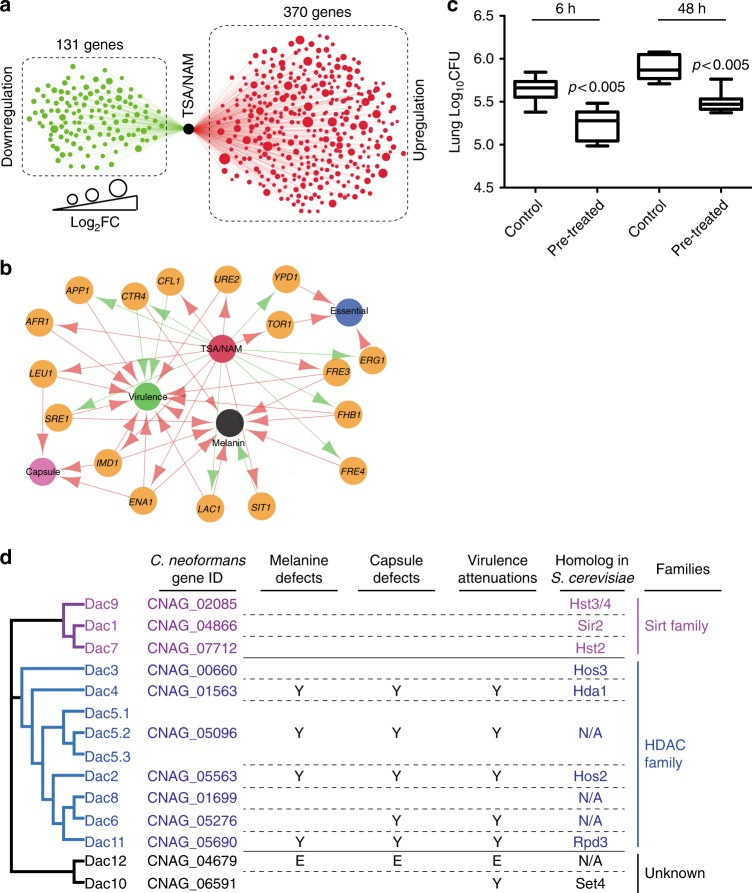
Kac is *C. neoformans* virulence determinant. **a** Transcriptome analysis of *C. neoformans* treated with trichostatin A (TSA)/nicotinamide (NAM). *C. neoformans* was treated with TSA/NAM. Green dots indicate repressed genes, and red dots indicate induced genes. **b** Regulatory network of virulence factors and deacetylase inhibitors. Red line indicates induction, and green line indicates repression. **c** Fungal burden of TSA/NAM-treated *C. neoformans*. Fungal cells were treated as described in Fig. 5a. *C. neoformans* cells were washed and subsequently infected in mice (*n* = 7). Colony-forming units from the lungs were assayed and plotted using box plot. Student’s *t* test was applied. **d** A phylogenetic analysis of deacetylases. Significant attenuations in virulence, melanin, and capsule were marked with Y. *DAC12* is an essential gene and is marked with E. Dac5.1–5.3 indicate alternative spliced proteins. N/A indicates no homolog in *S. cerevisiae*

Transcriptome analysis showed 131 repressed genes and 370 induced genes after treatment with the inhibitors (Fig. 5a, Supplementary Data 4). We noticed that some of the differentially expressed genes were associated with melanin formation, capsule production, and fungal virulence (Fig. 5b), suggesting that the inhibitors may influence fungal virulence in vivo. Next, we used a mouse infection model to examine fungal colonization after pretreatment of fungal cells with TSA/NAM. Because *C. neoformans* cells exhibited reduced growth after 12 h of incubation with TSA/NAM (Supplementary Fig. 5a), cells were preincubated with TSA/NAM for 6 h to avoid growth defects. Washed cells were intranasally infected in mice. Fungal burden analyses at 6- and 48-h post-infection demonstrated significant losses of TSA/NAM-treated fungal cells in these lung tissues (Fig. 5c).

In order to identify the mechanisms by which deacetylase inhibitors modulate fungal proliferation in the host, we annotated the *C. neoformans* genome for deacetylase-encoding genes. Twelve deacetylase genes were identified and designated *DAC1*–*12*. Gene knockout strains were constructed. Notably, *DAC12* is an essential gene (Supplementary Fig. 5b). Virulence features of each mutant were evaluated. While *dac2Δ*, *dac4Δ*, *dac5Δ*, and *dac11Δ* demonstrated impairments in melanin and capsule formation, *dac6Δ* displayed a capsule reduction phenotype (Supplementary Figs. 5c, d). In mice, five deacetylase knockout mutants exhibited lower virulence than the wild-type strain (H99); among these, *dac5Δ*, *dac6Δ*, *dac10Δ*, and *dac11Δ* showed modest attenuation in virulence and *dac2Δ* and *dac4Δ* showed significantly impaired fungal virulence (*p* < 0.001) (Fig. 3a and Supplementary Fig. 5e). A phylogenetic analysis indicates that Dac1, 7, and 9 have significant homology with the sirtuin family. Deacetylases that include Dac2–6, 8, and 11 have protein sequence similarities with the histone deacetylase (HDAC) family although Dac10 and Dac12 possess features found in deacetylase proteins but with minimal similarity to deacetylase proteins from other organisms, which suggests that they may be *C. neoformans* species-specific deacetylases (Fig. 5d). Interestingly, the majority of *C. neoformans* HDACs were found to modulate fungal virulence, indicating a unique pattern of regulation in HDACs compared to sirtuins (Fig. 5d). Our results demonstrate that Kac in *C. neoformans* in addition to the HDAC proteins, Dac2 and Dac4, are critical fungal fitness determinants.

### Dac2 and Dac4 are critical regulators for virulence

**Fig. 6:**
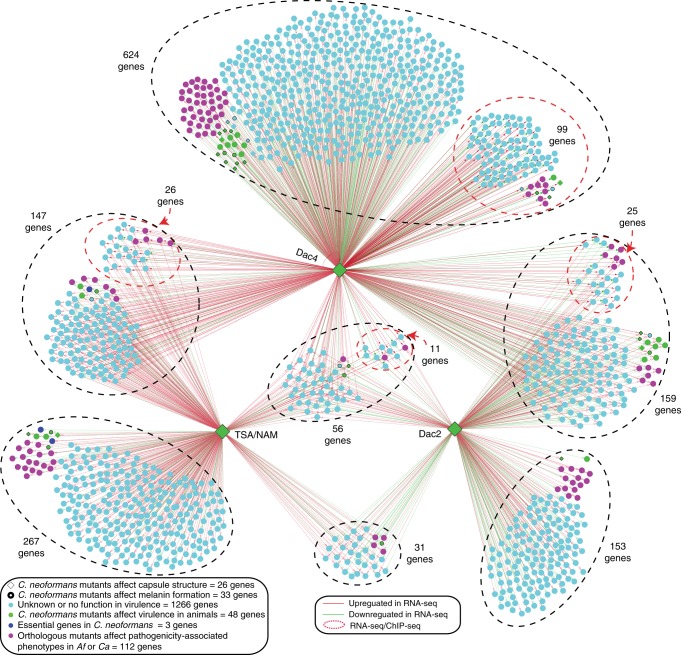
Dac2 and Dac4 regulatory network. The regulatory network in *dac2Δ*, *dac4Δ* and TSA/NAM-treated cells. The regulatory network was constructed using transcriptional profiling results in *dac2Δ*, *dac4Δ* and TSA/NAM-treated cells. Chromatin immunoprecipitation–sequencing (ChIP-seq) data in *DAC4-FLAG* strain was also integrated. Furthermore, known pathogenicity factors (including virulence, melanin, and capsule) from the literature were also integrated into the network. For uncharacterized *C. neoformans* genes, orthologs from *C. albicans* or *A. fumigatus* were sought in the literature for virulence modulation or pathogenicity-associated phenotypes (such as virulence, adhesion, filamentous growth, and invasive growth) and are shown in purple. Green represents known virulence factors in *C. neoforman*s. Light blue indicates unknown gene function in virulence. Black edges indicate genes involved in melanin. Diamonds show genes involved in the capsule phenotype in *C. neoformans*. ChIP-seq data are illustrated as red circles. Direct interactions (ChIP-seq data) are illustrated as solid lines, and dashed lines indicate indirect interactions. Upregulated genes are shown as red lines, and green lines indicate downregulated genes

As shown above, Dac2 and Dac4 are indispensable regulators for protein translation elongation process and fungal fitness in the host. An interesting question is whether Dac2 and Dac4 knockout mutants influence fungal fitness via elongation activity dampening, regulation of gene expression of virulence factors, or both. Our comparative acetylome analysis in *dac2Δ* and *dac4Δ* revealed that 19 histone Kac sites were robustly induced in *dac4Δ*, and 16 histone Kac sites were modestly increased in *dac2Δ* (Supplementary Data 1). These data suggest that the influence on *C. neoformans* pathogenicity by deacetylases may be via regulation of Kac levels in histones, which in turn alters gene expression of virulence factors. To test this hypothesis, the predominant histone Kac regulator (Dac4) was selected for chromatin immunoprecipitation–sequencing (ChIP-seq) analysis using the *DAC4-FLAG* strain (Supplementary Fig. 6a). While signals from the inputs remained low, the Dac4-Flag IP samples demonstrated strong binding at the promoter regions of target genes (Supplementary Fig. 6b, c, Supplementary Data 5). In order to determine the regulatory pattern, transcriptome analyses in *dac4Δ* cells were performed, verified and incorporated with the ChIP-seq data (Fig. 6 and Supplementary Fig. 6c, Supplementary Data 5). The transcriptome analysis in *dac2Δ* was included to compare with that found in *dac4Δ* (Supplementary Data 4). In addition, information from previous studies that examined virulence of *C. neoformans* and orthologs from *C. albicans* and *A. fumigatus* was also integrated (Supplementary Data 3) into this network. In the network, the expression of 56 genes was co-regulated by Dac2 and Dac4 and TSA/NAM, of which 11 genes were found to be directly regulated by Dac4 (Fig. 6, Supplementary Data 4 and 5). Of 56 genes, two genes (sodium transport ATPase, *ENA1* and ferredoxin reductase, *FRE3*) are known virulence determinants, and *FRE3* has been associated with melanin in *C. neoformans* (Fig. 6 and Supplementary Fig. 7, Supplementary Data 3). Three genes, whose functions are unknown in *C. neoformans*, were found to be pathogenicity regulators in *C. albicans* and *A. fumigatus* (Fig. 6, Supplementary Data 3). When comparing the data from Dac2 and Dac4, the expression of 159 genes was co-regulated by both deacetylases, and among these genes, the expression of 25 genes was directly regulated by Dac4 (Fig. 6, Supplementary Data 4 and 5). The expression of 267 genes was influenced by only TSA/NAM, and the expression of 153 or 624 genes were changed only when *DAC2* or *DAC4* was disrupted (Fig. 6). The gene expression of 10 virulence factors was co-regulated by Dac2 and Dac4 (Supplementary Fig. 7). The expression of 22 virulence genes was regulated by Dac4 only (Supplementary Fig. 7).

Collectively, the gene expression of 48 known virulence factors (including *DAC2* and *DAC4*), 33 factors affecting melanin, and 26 capsule determinants was differentially expressed when *C. neoformans* Kac was disturbed via disruption of *DAC2* or *DAC4* or in the presence of deacetylase inhibitors (Fig. 6 and Supplementary Fig. 7, Supplementary Data 3, 4 and 5). Furthermore, the expression of 112 uncharacterized *C. neoformans* genes, whose orthologous expression contributes to fungal virulence in *C. albicans* and *A. fumigatus*, was regulated by Dac2, Dac4, or TSA/NAM in *C. neoformans* (Fig. 6, Supplementary Data 3). These data demonstrate the regulatory importance of Dac2 and Dac4 and reveal extensive regulatory networks involved in fungal virulence. Taken together, the contribution of Dac2 and Dac4 to fungal fitness appears to occur via the regulation of protein synthesis and direct activation of the expression of important virulence factors. Notably, our results indicate potential functions of uncharacterized pathogenicity-associated genes in *C. neoformans*.

### Fungal pathogenicities coevolve with Kac site motifs

We have extensively demonstrated a significant correlation between Kac and fungal pathogenicity. However, given the limited conservation of acetylated proteins and Kac sites, whether the occurrences of Kac sites on virulence proteins are randomly selected during evolution and/or whether human fungal pathogens share common strategy in acetylation site selections has yet to be determined.

**Fig. 7:**
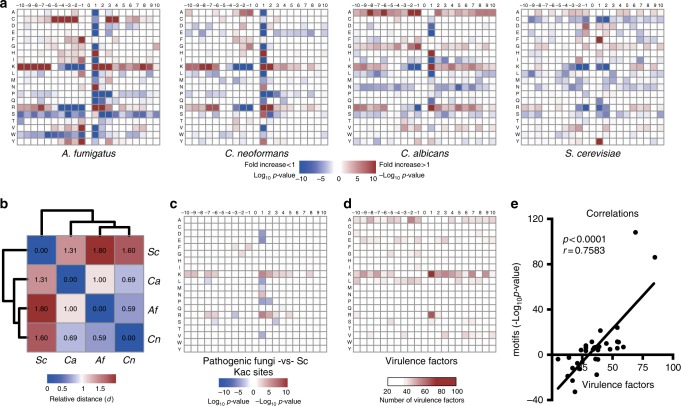
The analysis of Kac motifs. **a** Kac motif analysis of *C. neoformans*, *C. albicans*, *A. fumigatus*, and *S. cerevisiae*. Red squares indicate enriched amino acids; green or white squares suggest low possibility or unbiased occurrence of amino acids, respectively. **b** Relative distances of Kac motif layouts in four fungal species. The relative distances were calculated using the normalized root mean square distance. A heat map and distance tree was plotted. **c** Pathogen-specific motif enrichment. The significant Kac sites from Fig. 7a were combined. Enriched motifs (red and green squares) that over-represented in three pathogenic fungi, but either unenriched or shown as opposite enrichments in *S. cerevisiae*, were considered as pathogen-specific motifs. **d** Virulence factor counts. Reported and acetylome identified virulence factors from *A. fumigatus*, *C. albicans*, and *C. neoformans* were sorted based on Kac sites and plotted. **e** Correlation analysis between pathogen Kac motifs and virulence counts. Pearson’s correlation (*r*) tests and *p* value (two-tailed) were calculated

**Fig. 8:**
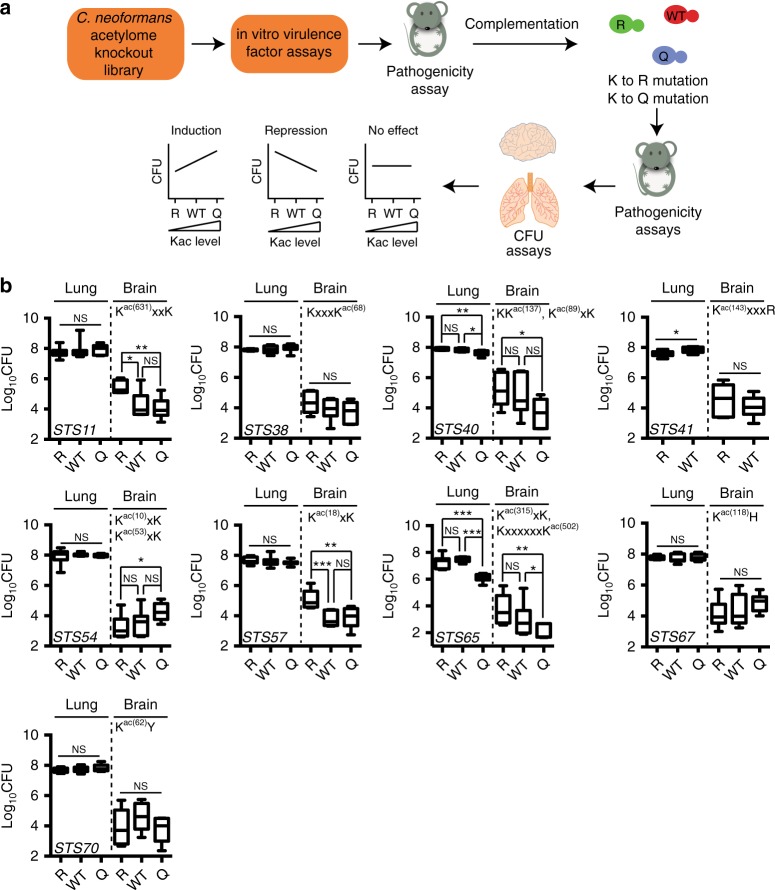
Pathogen-specific Kac motifs are critical regulators of fungal fitness in the host. **a** Screening strategy of Kac function in *C. neoformans* pathogenicity. Acetylome knockout library was constructed and screened for virulence attenuation. Knockout strains were then complemented with the wild-type, K-to-Q, and K-to-R mutated genes. The resulting strains were used for colony-forming unit (CFU) analysis. The CFU data were then plotted in an order of R-WT-Q, representing the level of protein acetylation. An increasing trend suggests an induction of virulence by Kac, a decreasing trend indicates a repression of virulence by Kac, and an unchanged trend shows no effect of acetylation in regulating virulence. **b** CFU analysis of mutation strains. Strains and mutated Kac sites are indicated. For each gene, the wild-type, Q, and R mutants were tested, except we could not obtain an *STS41*Q mutant complementation strain despite multiple attempts. CFUs from lungs (mice *n* = 7) were assayed and plotted using box plot. Student’s *t* test was applied

In order to address these questions, we analyzed the residue distributions flanking the acetylated lysine residues. Heat maps of residue distributions were generated, and this analysis demonstrated that residue distributions from pathogenic fungi somewhat resemble but differ from those of *S. cerevisiae* (Fig. 7a). A normalized root mean square distance analysis was performed in order to calculate the relative motif heat map similarities among the four species. The Kac heat map layouts from *C. albicans*, *A. fumigatus*, and *C. neoformans* share relatively short distances (≤1.0) with *S. cerevisiae* as an outgroup (Fig. 7b). By combining Kac motifs among pathogenic fungi and filtering those motifs from *S. cerevisiae*, a pathogen-specific Kac motif was generated, which showed that certain Kac motifs are strongly enriched in all pathogens such as K^ac^K, K^ac^xK, and K^ac^R (Fig. 7c). This may imply that these motifs have roles in virulence regulation. Known virulence factors from our acetylome data were also analyzed (Fig. 7d, Supplementary Data 3). A virulence distribution heat map was generated based on the Kac sites and the counts of published virulence factors. Interestingly, the virulence factor counts followed a strikingly similar pattern to that of the pathogenic-specific heat map and were strongly correlated (Fig. 7e), which implies that these sites may be functional in virulence regulation. To test this hypothesis, we generated a Kac knockout library, which was then complemented with a wild-type gene or K-to-R, or K-to-Q mutation (Fig. 8a). Colony- forming units (CFUs) were assayed to screen for Kac regulation of the changing fungal burdens. Greater numbers of CFUs with the Q mutation potentially indicate positive regulation by Kac on virulence factor function, and the opposite suggests possible repression. A non-significant change among the three strains suggests that Kac has no effect (Fig. 8a).

Without bias, we randomly disrupted 41 genes from the *C. neoformans* acetylome data and evaluated their potential roles in the expression of virulence factors in vitro and their fungal burdens in the lung and brain tissues (Supplementary Figs. 8 and 9). From these, 18 mutants were selected in order to further verify virulence in the host. Seventeen mutants demonstrated attenuation in virulence, except for *sts13Δ*, which showed no significant changes (Supplementary Fig. 9e). The knockout strains *sts65Δ*, *sts67Δ*, *sts70Δ*, *sts38Δ*, and *sts41Δ* were avirulent. Strains *sts26Δ* and *sts11Δ* were hyper-virulent compared to the H99 strains (Supplementary Fig. 9e).

Nine mutants were used for Kac regulation analysis. Our results showed that Kac mutations in Sts38 (hypothetical protein), Sts67 (hypothetical protein), and Sts70 (hypothetical protein) had no effect on fungal burdens. Kac mutations of Sts11 (nuclear protein), Sts54 (hypothetical protein), and Sts57 (transcription export THO complex) resulted in significant alterations in CFUs in the brain, and Sts41 (clathrin heavy chain) Kac mutant was reduced in the lung (Fig. 8b), indicating niche-favorable regulation by Kac. A K-to-Q mutation in Sts40 (Het-C2) and Sts65 (Ypk1) resulted in reduced CFUs in both the lung and brain. Assayed Kac sites, including K^ac^xxK, K^ac^xK, K^ac^xxxR, K^ac^K, and KxxxxxxK^ac^ of Sts11, Sts40, Sts41, Sts54, Sts57, and Sts65, which are pathogen-enriched motifs (Fig. 8b), function as regulators of fungal virulence, whereas Kac sites in Sts67, Sts38 and Sts70, including KxxxK^ac^, K^ac^H, and K^ac^Y, which are not enriched in pathogens (Fig. 7c), were not involved in this regulation. These data suggest that enriched pathogen-specific Kac motifs are involved in the regulation of fungal pathogenicity via a non-epigenetic mechanism. The data also imply that there has been positive selection pressure on Kac motifs in fungal pathogens during evolution.

## Discussion

Fungal species are rapidly evolving organisms that have developed sophisticated and complicated regulatory mechanisms to battle harsh environments^5,11,13^. The current analysis of fungal evolution is focused primarily on the genomic level, where gains or losses of genes are analyzed. For example, a comparative genomic analysis demonstrates that virulence-associated genes in human pathogens have extensively expanded in many important processes in the genome, and these include cell wall formation and maintenance, transporter genes, protein secretion, and metal homeostasis in addition to genes involved in pathogen adaptation, colonization, and replication in the host^5^. Fungal cells respond to environmental stimuli via rapid modulation of transcriptional regulation. Lysine acetylation is an essential linkage in this transcriptional alteration via histone modification. Additionally, non-histone protein activities, which contribute to important stress-related cell adaptation, are also regulated by acetylation^3,4^. However, the way in which the acetylation regulatory machinery evolves in various organisms is unknown.

In this study, an interspecies analysis unveiled the evolutionary pattern of fungal acetylomes. Data analyses across four fungal species revealed two types of Kac evolutionary mechanisms: (1) orthologous Kac selection and (2) specific Kac site selection. Only orthologs from the core biological processes were conserved in the acetylation regulatory mechanism despite limited detection of conservation in Kac sites, demonstrating a rapidly evolving rate in Kac site selections by various fungal species. A closer look at the Tef1 orthologs supports the hypothesis that Kac sites within critical domains tend to be conserved. Additionally, Kac sites were detected in the GTP-binding domain of Tef1 for all tested fungal species. These results demonstrate a novel regulation mechanism for the eukaryotic elongation process and conceptually suggest that, although Kac modification of orthologous proteins in central biological processes is conserved, species-distinct Kac sites that fit into individual life cycles can be found in distantly related fungi.

Further analysis of the Tef1 Kac regulatory machinery demonstrates that Dac2 and Dac4 act as non-epigenetic regulators (via acetyl group erasure) for protein synthesis machineries. Both deacetylases share common non-histone client proteins, and of these, Dac2 and Dac4 contribute to the modulation of Tef1 Kac via direct protein–protein interactions. The K-to-Q mutation of Kac sites in the GTP-binding domain of Tef1 led to severe growth defects, and the Kac levels of Tef1 were strongly induced when disrupting *DAC2* or *DAC4* genes, suggesting that the deacetylation process is essential for Tef1’s function, and Dac2 and Dac4 act as GTP-loading determinants in Tef1 in a functionally redundant manner. Given the confirmation of Kac in Tef1 orthologs from other fungal species and mammalian cells, a conserved regulatory machinery is suggested for protein synthesis by Kac. In fact, Hu et al. suggested the Kac regulation phenomenon in translation elongation factor 1A (eEF1A, a Tef1 homolog) in tumor cells and demonstrated that HDACs, rather than sirtuins, contribute to the regulation of Kac levels^25^. Considering previous results, our data extensively verify that the regulation of Tef1 Kac by HDAC is a highly conserved machinery from fungi to mammals. It is therefore interesting to test whether Dac4 and Dac2 homologs in human cells share the same regulatory role in eEF1A activity. Importantly, previous studies suggest that HDAC inhibitors are potential clinical treatments for fungal infections^20,22^. However, given the essential role of Kac in Tef1 activity and the similar regulation of Tef1 Kac between fungi and the host, specifically targeting fungal deacetylases remains a hindrance in the development of antifungal treatments.

Our analysis provides the evidence of a fast-evolving Kac rate in major human fungal pathogens. But, what is the driving force for this rapid evolution of fungal Kac site selection? One possible explanation relies on the evolutionary rate of important enzymes in Kac. Analyzing *C. neoformans* deacetylase knockout strain phenotypes reveals that *C. neoformans* HDAC proteins play more predominant roles in controlling virulence than do sirtuin proteins. These data elucidate the evolutionary advantage of HDAC over sirtuin proteins during system infection. Although previous work demonstrated various virulence-associated phenotypes of *dac1Δ* (*sir2Δ*) in murine infections^26^, our data demonstrate that the function of *C. neoformans* sirtuin homologs in fungal virulence regulation is null. The difference in *dac1Δ* strains is most likely the result of rapid microevolution in H99 strains, in which epigenetic regulation of *C. neoformans* pathogenicity is likely reprogrammed^27^.

Studies demonstrate that Dac4 (Hda1) participates in controlling cell morphology in *C. albicans* and form a polycomb repression complex in *C. neoformans*^28,29^. Recently, Brandão et al. revealed the roles of Dac4 in regulating heat shock response, melanin formation, capsule structure, and virulence in *C. neoformans*^30^. Interestingly, *C. albicans* Hos2 (Dac2) has a function reciprocal to Hda1 in regulating cell morphology^31^, whereas its function in *Candida* pathogenicity remains uninvestigated. Unlike *C. albicans*, but consistent with *C. neoformans* data, we demonstrated that Dac2 and Dac4 co-regulate various biological processes and are predominant positive regulators of virulence factor production (via epigenetic modification of Kac levels) in *C. neoformans* histones. Dac2 and Dac4 balance the gene expression of many critical *C. neoformans* virulence factors and appear to be characteristic of virulence orthologs from *A. fumigatus* and *C. albicans*. Additionally, the function of *C. neoformans* deacetylases showed species-specific phenotypes compared those from other fungi. For example, although Rpd3 has an important function in *S. cerevisiae* chromatin remodeling and white-opaque switching in *C. albicans*^29,32,33^, the *C. neoformans* ortholog (Dac11) is a minor participant in *C. neoformans* pathogenicity, melanin formation, and capsule production. Taken together, given that fungal life cycles are dramatically divergent, it is most likely that these deacetylases have undergone significant rewiring in modulating client protein, thus resulting in fast-evolving Kac events in fungi.

Despite the limited conservation in Kac sites in pathogenic fungi, our results showed that a large number of pathogenicity-associated factors are acetylated proteins, indicating that their functions are potentially influenced by Kac. Clearly, the environmental conditions are the key driving forces for acetylation selection and evolution. Given that these three fungi are successful pathogens that can survive and replicate in harsh host conditions, is the host niche the critical influencer behind the Kac divergence, and do pathogens share common strategies for Kac selection? We show that Kac motifs in pathogenic fungi drastically differ from those in *S. cerevisiae*, demonstrating that pathogen-favorable Kac sites are beneficial to fungal fitness in host niches. In agreement with this hypothesis, pathogen-enriched Kac motifs were shown to participate in regulating fungal colonization in tissues. In contrast, Kac motifs that are not enriched in pathogens have no role in the control of fungal pathogenicity in the host.

In conclusion, interspecies acetylome comparison analysis revealed divergence and highly dynamic features of Kac modifications within fungal species. Using an integrative omics analysis, we have elucidated the deacetylases’ molecular mechanisms that are required for essential biological processes in addition to virulence expression modulation in *C. neoformans*. The activities of crucial and conserved processes were shown to be manipulated by Kac. Despite the unfavorable conservation of Kac sites in fungi, common Kac motifs are shared by major human fungal pathogens, suggesting an indispensable role for these motifs in their contribution to the fungal life cycle during infection. Therefore, our data illustrate the conserved regulatory machinery that is beneficial for understanding fungal pathogenicity and its fungal evolution. Most importantly, our results call for a thorough evaluation of acetylome evolution in higher organisms.

## Methods

### Strains and media

Fungal cells (Supplementary Data 7) were routinely grown in YPD medium (1% yeast extract, 2% peptone, 2% dextrose). For biolistic transformation, YPD medium supplemented with 100 μg/ml nourseothricin (WERNER BioAgents), 200 μg/ml neomycin (Inalco), and 200 U/ml hygromycin B (Calbiochem) was used for colony selection. Three micromolar TSA and 20 mM NAM (Sigma) were used for blocking deacetylases. L-DOPA agar medium was used for melanin formation. Dulbecco’s Modified Eagle Medium (DMEM), supplemented with 10% of fetal bovine serum (FBS) was used for capsule production in *C. neoformans* and hyphal formation in *C. albicans*. YPGal medium (1% yeast extract, 2% peptone, 2% galactose) was used to test *GAL7-TEF1* and *GAL7-DAC12* strains. YUU medium (0.5% yeast extract, 0.1% v/v trace elements, 2% glucose, 0.12% uridine, 0.11% uracil) was used for *A. fumigatus* transformation.

### Generation of fungal mutants

All primers used in the generation of fungal mutants are listed in Supplementary Data 8. The *C. neoformans* mutants were generated using the H99 strain and biolistic transformation^34,35^. Briefly, the upstream or downstream genomic DNA sequences of target genes were amplified using the primers listed in Supplementary Data 8. The neomycin or nourseothricin resistance marker was amplified using primers 23 and 24. The upstream and downstream DNA sequences of the target gene and the selective marker sequence were joined by overlapping PCR. The resulting PCR fragments were purified, concentrated and transformed into the H99 strain using biolistic transformation. Transformants were selected on YPD agar supplemented with neomycin or nourseothricin. Correct integration and the loss of target DNA sequences were confirmed using diagnostic PCR and real-time PCR.

For the generation of the complementation strains, the complete open reading frame (ORF) of the target gene (including the promoter sequence) was amplified and cloned into the pTHYG plasmid (a plasmid containing the terminator sequence from *GPD1* and the hygromycin B resistance marker), generating the wild-type gene complementation plasmid. The resulting plasmid was used for the generation of the R or Q mutant plasmid using the TaKaRa MutanBEST Kit (Takara). The downstream DNA sequence of the target gene was then joined by overlapping PCR. The resulting PCR products were transformed into knockout strains. Complementation was confirmed by diagnostic PCR and real-time PCR. The R and Q mutants were further confirmed by DNA sequencing.

The *STS65*-*FLAG* complementation strains were generated as follows. First, a p4FlagHYG plasmid was generated. Primers 1717 and 1718 were used to amplify a hygromycin resistance marker, introducing a 4× *FLAG* DNA sequence before the *GPD1* stop codon. The upstream genomic DNA sequence of *STS65* was amplified using primers 1866 and 1867 and cloned between the restriction sites *Xho*I and *Hind*III. For the R mutation, two rounds of mutation PCR and ligation were performed. The K315 codon was first mutated using primers 3116 and 3117, and then the K502 codon was mutated using primers 1869 and 1868. The resulting plasmid contained the *STS65R* mutations (K315R and K502R). The downstream sequence of *STS65* was ligated using overlapping PCR to generate the integration cassette. For the Q mutation, the same procedures were carried out, except that the primer pairs 3118/3117 and 2380/1868 were used to generate K315Q and K502Q. The wild-type, R mutant, and Q mutant integration cassettes were integrated separately into the *sts65Δ* strain. The gene complementation was confirmed using diagnostic PCR, real-time PCR, and immunoblotting.

To investigate the regulation of Tef1 by Kac, we generated a *GAL7-TEF1* strain, in which the expression of *TEF1* is driven by galactose and repressed by glucose. The construct *TEF1* upstream-*NEO*-*GAL7-TEF1ORF* was generated, which allows integration to occur at the *TEF1* locus to replace the *TEF1* native promoter with a *GAL7* promoter. Briefly, the *TEF1* upstream genomic DNA sequence was amplified using the primers 3314 and 3315 and cloned into the pNEO plasmid between the *Apa*I and *Xho*I sites. The genomic DNA sequence of the *GAL7* promoter was amplified using primers 3316 and 3317 and then fused with the *TEF1* ORF by overlapping PCR. The resulting fragment was cloned into the above plasmid between *Bam*HI and *Sac*II. The plasmid was PCR-amplified and transformed into the H99 strain, generating the *GAL7-TEF1* strain. To complement the *GAL7-TEF1* strain with wild-type or mutated *TEF1*, a construct, *CMT2* upstream-*NAT*-*TEF1* promoter-*TEF1* ORF (WT or mutant)-*2XFLAG*-*3’UTR-CMT2* downstream, was generated to allow integration at the *CMT2* locus and to produce Tef1-Flag. The *TEF1* Kac mutation genes were constructed using overlapping PCR. For the R mutation, three DNA fragments were amplified using the primer pairs 3301/3306 and 3357/3309 (generating K36R and K41R) and 3359/3304 (generating K217R). The resulting fragments were joined by overlapping PCR. For the Q mutation, a similar protocol was followed, except that the primer pairs 3301/3306, 3358/3309, and 3360/3304 were used.

To prove the direct protein–protein interaction of Tef1 and deacetylases, the DNA sequences of *DAC2* or *DAC4* were amplified and cloned into the plasmid pHA-HYG. The resulting plasmids were transformed into the strain *GAL7-TEF1 CMT2::TEF1W*. The strain *TEF1-HA* was generated by integrating the pTEF1-2HA plasmid into the native allele of *TEF1* to generate *TEF1-HA* with expression driven by its native promoter. The genomic DNA sequences of *DAC2* or *DAC4* were amplified and cloned into the plasmid p4XFLAG-NAT, which was transformed into the strain *TEF1-HA*.

The *C. albicans TEF1-FLAG* strain was generated using the SN152 strain and pNIM1 plasmid. The open reading fragment of *TEF1* was PCR amplified from *C. albicans* genomic DNA using the primers 3570 and 3571, and the purified fragment was joined with *Sal*I*-*digested pNIM1-His (harboring a *CaHIS1* selection marker) using the In-Fusion HD Cloning Kit (Clontech). The resulting plasmid was transformed into the SN152 strain. The expression of Tef1-Flag was then confirmed using immunoblotting.

The *S. cerevisiae TEF1-FLAG* strain was generated using the BY4741 strain and pGPD426 plasmid. The *TEF1* ORF was amplified from the genome using primers 3578 and 3579, introducing a DNA sequence encoding *FLAG* and two restriction sites (*Sal*I and *Xho*I). The digested PCR fragment was cloned into the pGPD426 plasmid, and the expression of *TEF1-FLAG* was driven by a constitutive promoter. The cloned plasmid was then transformed into the *S. cerevisiae* BY4741 strain. The expression of Tef1-Flag was confirmed using immunoblotting.

The *A. fumigatus TEF1-FLAG* strain was generated using the A1160 strain. The Af*TEF1* gene was amplified using the primers 3850 and 3851. The *pyr4* gene was amplified using the primers 3852 and 3855, introducing a 5× *FLAG* DNA coding sequence. The two resulting PCR fragments were joined by overlapping PCR, and transformation was performed in the A1160 strain. The expression of AfTef1-Flag was confirmed by immunoblotting.

### Antibodies

Mouse anti-acetyllysine primary antibody (clone Kac-01), PTM Bio, Cat No. PTM-101; Rabbit anti-acetyllysine primary antibody, PTM Bio, Cat No. PTM-105; Rabbit anti-HA primary antibody, Abcam, Cat No. AB9110; Rabbit anti-FLAG primary antibody, Abcam, Cat No. AB1162; Rabbit anti-Histone H3 (D1H2) primary antibody, Cell Signaling Technology, Cat No. 4499; Goat anti-Mouse IgG (H+L) Secondary Antibody, Invitrogen, Cat No. 31430; and Goat anti-Rabbit IgG (H+L) Secondary Antibody Invitrogen Cat No. 31460 were used.

### Acetylome analysis

Overnight cultures of *C. neoformans* H99 were diluted in fresh YPD media and incubated at 30 or 37 °C for 6 h. For the analysis of the Kac levels in *Cndac2Δ* and *Cndac4Δ*, mutant cells and wild-type cells were subcultured at 30 °C, and cells in the exponential phase were used. Comparative acetylome measurements in *C. neoformans* H99, *dac2Δ*, and *dac4Δ* were performed in biological triplicates. All acetylome results were pooled to obtain the *C. neoformans* acetylome. *C. albicans* (SC5314) cultures were diluted in YPD media at 30 °C for 6 h or DMEM (10% of FBS) at 37 °C with 5% CO_2_ for 6 h. Acetylome measurements of the pooled samples of *C. albicans* yeast and hyphae cells were performed in biological triplicates. *A. fumigatus* (Af293) cultures were diluted in fresh YPD media and incubated at 30 or 37 °C until cell density reached the exponential phase. Proteins samples isolated from cells grown at 30 or 37 °C were pooled. Acetylome measurements in *A. fumigatus*, grown at 30 and 37 °C, were performed in biological triplicates.

Cells were subsequently washed twice in phosphate-buffered saline (PBS) buffer supplemented with TSA/NAM. Cell pellets were resuspended in acetylome lysis buffer (8 M urea, 1% Triton X-100, 10 mM dithiothreitol, 1% protease inhibitor cocktail, 3 μM TSA, 50 mM NAM, and 2 mM EDTA), and suspensions were sonicated three times on ice using a high-intensity ultrasonic processor (Scientz). Protein samples were precipitated using TCA, resuspended in 8 M urea solution, and quantified using a BCA assay. Protein samples were treated with dithiothreitol (5 mM) and iodoacetamide (11 mM), diluted, and digested with trypsin at 37 °C overnight. Digested protein samples were desalted and vacuum dried. The peptides were resuspended in 0.5 M TEAB and processed for the 6-plex TMT Kit according to the manufacturer’s instructions. Briefly, one unit of TMT reagent (defined as the amount of reagent required to label 1.8 mg of peptides) was reconstituted in 80 μl acetonitrile buffer. The peptide mixtures were then incubated for 2 h at room temperature and pooled, desalted, and dried. The labeled peptides were then fractionated using an HPLC with a BetaSil C18 column (5 μm film thickness, 10 mm × 250 mm; Thermo Fisher Scientific, USA). Fractionated peptides were resuspended in immunoprecipitation (IP) buffer (100 mM NaCl, 1 mM EDTA, 50 mM Tris-HCl, 0.5% NP-40, pH 8.0). The suspended samples were then mixed with anti-Kac pan antibody-conjugated agarose beads (PTM Bio, China) and incubated at 4 °C overnight. The beads were washed 4 times with IP buffer and 2 times with water and eluted 3 times with 0.1% trifluoroacetic acid solution (Sigma, USA). The samples were desalted according to the C18 ZipTips manufacturer’s instructions^36^.

The peptides were dissolved in 0.1% formic acid and loaded onto a homemade reversed-phase analytical column (15 cm in length, 75 μm in diameter). The gradient was composed of an increase from 6 to 23% in 0.1% formic acid in 98% acetonitrile over 26 min, 23–35% in 8 min, and climbing to 80% in 3 min then holding at 80% for the last 3 min, at a constant flow rate of 400 nl/min on an EASY-nLC 1000 UPLC system. The peptides were then subjected to an NSI source, followed by tandem mass spectrometry (MS/MS) in a Q Exactive Plus (Thermo Fisher Scientific, USA) coupled online to the UPLC. The electrospray voltage was 2.0 kV. The *m*/*z* scan range was 350–1800 for full scan, and intact peptides were detected in the Orbitrap at a resolution of 70,000. Peptides were then selected for MS/MS using an NCE setting of 28, and the fragments were detected in the Orbitrap at a resolution of 17,500. A data-dependent procedure that alternated between one MS scan and 20 MS/MS scans with 15 s dynamic exclusion was performed. Automatic gain control was set at 5 × 10^4^ ^36^.

The resulting MS/MS data were processed using the MaxQuant search engine (version 1.5.2.8). Tandem mass spectra were searched against the UniProt database (http://www.uniprot.org) that was concatenated with a reverse decoy database. Trypsin/P was specified as a cleavage enzyme, and up to four missing cleavages were allowed. The mass tolerance for precursor ions was set at 20 ppm in the first search and 5 ppm in the main search, and the mass tolerance for fragment ions was set as 0.02 Da. Carbamidomethyl on Cys was specified as a fixed modification, and acetylation modification and oxidation on Met were specified as variable modifications. False Discovery Rate was adjusted to <1%, and the minimum score for modified peptides was set as >40^36^.

For the analysis of differentially expressed Kac in *Cndac2Δ* and *Cndac4Δ*, whole-proteome analysis was also performed as described above (except for the antibody enrichment step). The acetylome data of *Cndac2Δ* and *Cndac4Δ* were then normalized to the proteome data.

### Transcriptome analysis

The transcriptome analysis was performed as previously described, with modifications^37^. For the analysis of deacetylase inhibitors, *C. neoformans* cells were treated or not treated with 3 μM TSA and 20 mM NAM in 50 ml of YPD media at 30 °C for 6 h. For the analysis of dac2Δ and dac4Δ, overnight cell cultures of the wild-type H99, dac2Δ, and dac4Δ strains were subcultured in fresh YPD media, and the cultures were incubated at 30 °C until the cell densities reached the exponential phase (approximately 6–7 h). Cells were then washed twice with ice-cold PBS supplemented with 3 μM TSA and 20 mM NAM, followed by RNA isolation using TRIzol reagent (Thermo Fisher Scientific). Three micrograms of total RNA were processed using the TruSeq RNA Sample Preparation Kit (Illumina), and mRNA purification was performed using polyT oligo-attached magnetic beads. mRNA fragmentation was performed using an Illumina proprietary fragmentation buffer. First-strand cDNA was synthesized using random hexamer primers and SuperScript II. Later, second-strand cDNA was synthesized using RNase H and DNA polymerase I. Adenylation at the 3′ end of the cDNA sequence was carried out. cDNA sequences with 200 bps were used for the AMPure XP system (Beckman Coulter) and enriched using Illumina PCR Primer Cocktail in a 15-cycle PCR. The resulting PCR products were then purified, and the integrity was confirmed using the Agilent High Sensitivity DNA assay on a Bioanalyzer 2100 (Agilent). The sequencing library was then processed using a NextSeq 500 platform (Illumina). TopHat (http://ccb.jhu.edu/software/tophat/) was used to map the reads to the genome. RPKM was used for data normalization, and Cufflinks (http://cufflinks.cbcb.umd.edu) was used to assemble the transcripts^38^. Differentially expressed transcripts were analyzed using DESeq, as previously described^39^. Genes with adjusted *p* values <0.05 and fold changes greater or less than 1.5 than that of the control strain were considered to be significantly induced or repressed, respectively.

### ChIP-seq analysis

The ChIP assay was performed using the ChIP-IT High Sensitivity Kit (Active Motif, Carlsbad). The DAC4-FLAG strain cells were diluted in fresh YPD media. Cells were fixed with 1% formaldehyde (Sigma) for 15 min and quenched using 125 mM glycine. Cells were washed three times with ice-cold PBS buffer supplemented with 125 mM glycine. Cells were then lysed with lysis buffer (10 mM Tris-HCl, 10 mM NaCl, 3 mM MgCl2, 0.5% IGEPAL). Cell pellets were suspended in sonication buffer (20 mM Tris-HCL (PH 8.0), 2 mM EDTA, 1% Triton-X100, 150 mM NaCl, 1% sodium dodecyl sulfate (SDS), 100 µM phenylmethanesulfonylfluoride (PMSF), and 1× proteinase inhibitor). The samples were sheared with a Diagenode Pico device using 30 s on and 30 s off for 30 cycles. After centrifugation, the supernatant was collected and diluted in IP dilution buffer (20 mM Tris-HCl, 2 mM EDTA, 1% Triton X-100, 150 mM NaCl, 100 μM PMSF, and 1× proteinase inhibitor). IP was performed with 5 μg anti-Flag ChIP grade antibody and Protein G Beads at 4 °C overnight. The beads were then washed four times in IP dilution buffer. The sample was then eluted with elution buffer and treated with Proteinase K overnight at 65 °C. DNA samples were purified with DNA purification columns. The ChIP-seq library was constructed using the MicroPlex Library Preparation Kit v2 (Diagenode) according to the manufacturer’s instructions. The ChIP DNA libraries were sequenced using an Illumina HiSeq 2500 Platform. Raw reads were mapped to the *C. neoformans* H99 genome (downloaded from http://fungidb.org/fungidb/) using the Bowtie 2 suite (version 4.1.2)^40^. The SAM files were then sorted using SAMtools (version 1.3.1)^41^. The ChIP-seq peaks were analyzed using the MACS suite (Model-based analysis of ChIP-seq, version 1.4.2) with a *p* value cutoff of 10^−8 ^^42^. The ChIP-seq signal was visualized using IGV (Integrative Genomic Viewer, version 2.3.98)^43^.

### Kac motif analysis

The model of sequences constituting with amino acids in specific positions of modify-21-mers (10 amino acids upstream and downstream of the Kac site) in all protein sequences were downloaded at www.uniprot.org and analyzed. Statistical significance for the enrichment of Kac motifs were calculated using Fisher test. In addition, all the database protein sequences were used as the background database parameter, and the other parameters remained as defaults.

### Ethics statement

All animal experiments were carried out under the review and approval of the Research Ethics Committees at the College of Life and Health Sciences of Northeastern University. *C. neoformans* infections were performed via the intranasal route. Six-to-8-week-old female C57BL/6 mice were purchased from Changsheng Biotech (Liaoning, China) and used for survival and fungal burden analysis^44^. All animal experiments were carried out in accordance with the regulations in the Guide for the Care and Use of Laboratory Animals issued by the Ministry of Science and Technology of the People’s Republic of China.

### Animal infection

For the fungal burden analysis, mice were anesthetized and intranasally administered 10^5^ fungal cells in 50 μl of PBS buffer. Infected mice were sacrificed on post-infection day 14. Lung or brain tissues were removed, weighed, and homogenized in 5 or 1 ml of PBS buffer, respectively. The homogenized suspension was diluted and plated onto YPD agar plates, which was incubated at 30 °C for 2–3 days. The animal survival analysis was performed as described in the fungal burden assay. Infected animals were monitored twice a day for morbidity.

### Protein IP and immunoblotting analysis

Cell proteins were isolated using a Mini-BeadBeater-16 (BioSpec) and lysis buffer (50 mM Tris-HCl, 150 mM NaCl, 0.1% NP-40, pH 7.5), supplemented with 1× protease inhibitor cocktail (CWBIO), 40 mM PMSF, 3 μM TSA, and 20 mM NAM. Aliquots of protein lysis solution were saved as input samples. Three-microgram lysed protein samples were incubated with Anti-FLAG M2 Magnetic beads (Sigma) at 4 °C for 4 h. For the detection of protein acetylation, beads were washed four times with TBS buffer (50 mM Tris-HCl, 150 mM NaCl, 1% Triton X-100, pH 7.4), supplemented with 3 μM TSA and 20 mM NAM. For the detection of protein–protein interaction, TBS buffer without Triton X-100 was used for washing.

The bound protein molecules were eluted in protein loading buffer by boiling at 95 °C for 3 min. The eluted protein samples were separated using 12% SDS–polyacrylamide gel electrophoresis transferred onto nitrocellulose membrane, and blocked with 5% milk. Immunoblotting assays were performed using anti-M2 Flag (1:5000, Abcam), anti-HA (1:5000, Abcam), anti-Histone H3 (1:5000, Cell Signaling Technology), monoclonal and polyclonal Kac (1:2500, PTM Bio), and anti-mouse and anti-rabbit (1:5000, Thermo Fisher Scientific) antibodies. The signal was captured using a ChemiDoc XRS+ (Bio-Rad).

### Ortholog analysis and network construction

Orthologs of differentially expressed genes and acetylated proteins were searched for in FungiDB (http://fungidb.org/fungidb/)^45^ and OrthoMCL (http://orthomcl.org/orthomcl/)^46^. The thresholds of OG5 groups were defined as *E*-value < 1e^−5^ (Log_10_^(*E*-value)^ < −5) when percent match length was at least 50% based on OrthoMCL. The conserved Kac sites were determined from ortholog multiple alignment using Clustal Omega (https://www.ebi.ac.uk/Tools/msa/clustalo/). Aligned K residues that are detected in our acetylome analysis are considered as conserved Kac sites. The regulatory networks of differentially expressed genes or acetylated proteins were constructed using Cytoscape (version 3.6.0)^47^. Information regarding the functions of identified genes or proteins in the regulation of fungal virulence were obtained from published literature (https://www.ncbi.nlm.nih.gov/pubmed).

### Statistics and reproducibility

The statistical test Log-rank (Mantel–Cox) was used for the animal survival tests. Paired-sample *t* test was used in two-sample comparisons. Pearson’s correlation (*r*) tests were performed in Prism 6. Significant changes were defined as *p* < 0.05. *p* values are indicated in the plots or represented as ${}^{\ast \hskip -1pt}$p < 0.05, ${}^{\ast\ast \hskip -1pt}$p < 0.01, or ${}^{\ast\ast\ast \hskip -1pt}$p < 0.005. All experiments were performed using three biological replicates to ensure the reproducibility.

Significance of the Kac motifs were calculated using Fisher test. The relative relationships (distances) among the motifs were determined using the normalized root mean square distance.

$$d = \frac{{\mathop {\sum }\nolimits_{u = 1}^N \mathop {\sum }\nolimits_{v = 1}^N \left( {t_{u,v} - r_{u,v}} \right)^2}}{{\mathop {\sum }\nolimits_{u = 1}^N \mathop {\sum }\nolimits_{v = 1}^N \left( {\left( {\left( {t_{u,v} - {\bar{t}}} \right)^2 + \left( {r_{u,v} - {\bar{r}}} \right)^2} \right)/2} \right)}}$$

where *d* is the sum of the relative distance between the Kac motifs of any two fungal species, and *t*_*u,v*_ and *r*_*u,v*_ represent the pixel values of any given Kac motifs of two fungal species.

### Reporting summary

Further information on research design is available in the Nature Research Reporting Summary linked to this article.

## Supplementary information

Supplementary Information

Supplementary Data 1

Supplementary Data 2

Supplementary Data 3

Supplementary Data 4

Supplementary Data 5

Supplementary Data 6

Supplementary Data 7

Supplementary Data 8

Description of Supplementary Data

Reporting Summary

## Data Availability

The raw acetylome and proteome mass spectrometric data have been deposited to the ProteomeXchange (https://www.ebi.ac.uk/pride) with identifier PXD010354. The transcriptome (RNA-seq) and ChIP-seq data are deposited in NCBI’s Gene Expression Omnibus (GEO) (https://www.ncbi.nlm.nih.gov/geo/) and can be accessed through GEO Series accession ID GEO: GSE116040. Full immunoblot images corresponding to those presented in the main figures are available as Supplementary Fig. 10. Any other data necessary to support the conclusions of the study are present in the supplementary data files or available from the authors upon reasonable request.
