# Supplementary material for: Fungal acetylome comparative analysis identifies an essential role of acetylation in human fungal pathogen virulence
Source: Commun Biol. 2019 May 1;2:154. doi: 10.1038/s42003-019-0419-1 (PMC6494858; doi:10.1038/s42003-019-0419-1)
Supplement: Supplementary file 1 — Supplementary Information [file 42003_2019_419_MOESM1_ESM.pdf]

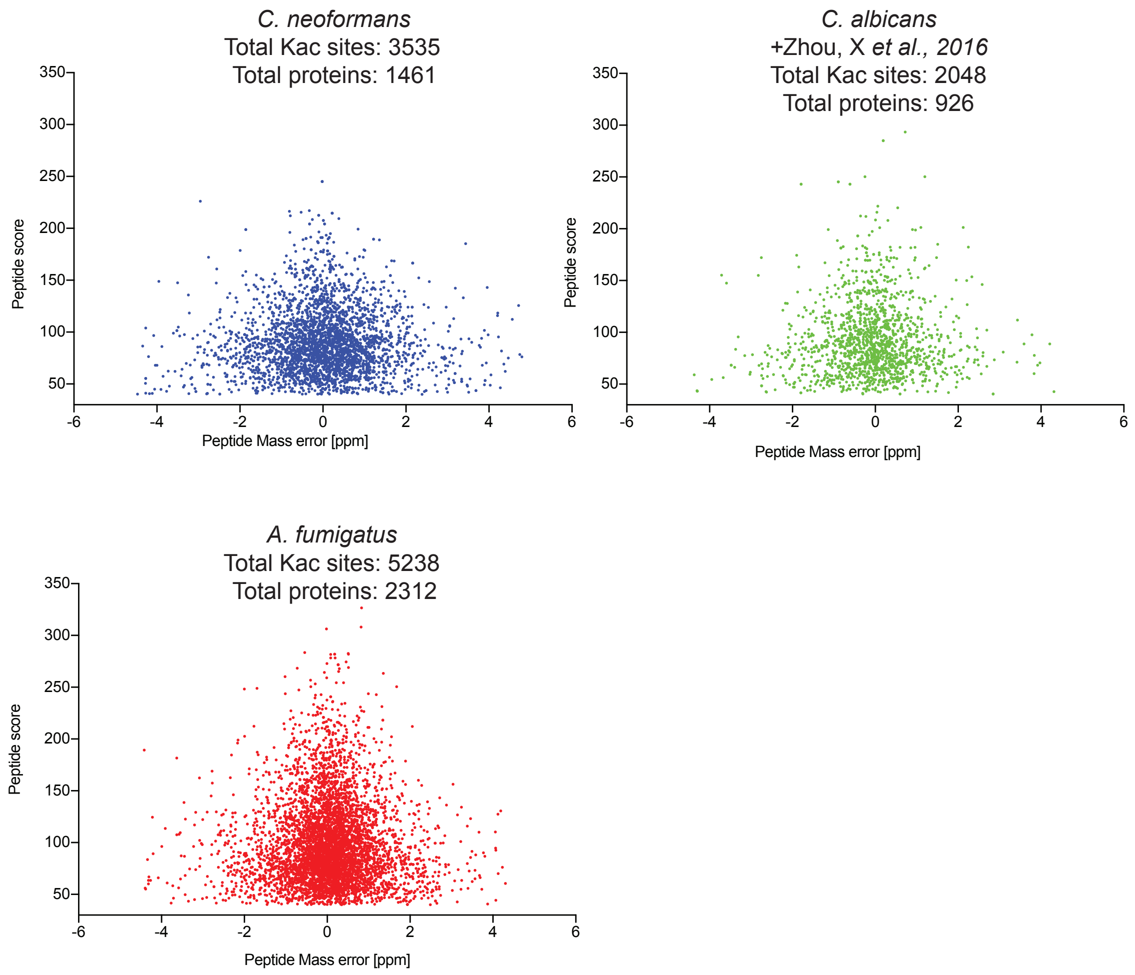

Supplementary Figure 1. Acetylome analysis in pathogenic fungi.

The mass errors of identified Kac peptides in *C. neoformans*, *C. albicans* and *A. fumigatus*. *C. neoformans* acetylome data contains the acetylome data that include three biological replicates from *dac2Δ*, three biological replicates from *dac4Δ,* and three biological replicates from the H99 strain and also includes two additional replicates from the H99 strains grown at 30°C or 37°C. In total, 3535 Kac sites (1461 proteins) were identified in *C. neoformans*. *A. fumigatus* acetylome was created using three biological replicates and pooled protein samples isolated from A293 cells grown at 30°C or 37°C. In total, 5238 Kac sites (2312 Kac proteins) were detected in *A. fumigatus*. *C. albicans* acetylome was created using three biological replicates and pooled protein samples isolated from yeast and hyphal cells. Our data were pooled with the *C. albicans* data from Zhou *et al.,* 2016[23]. In total, 2048 Kac sites (926 Kac proteins) were identified in *C. albicans*.

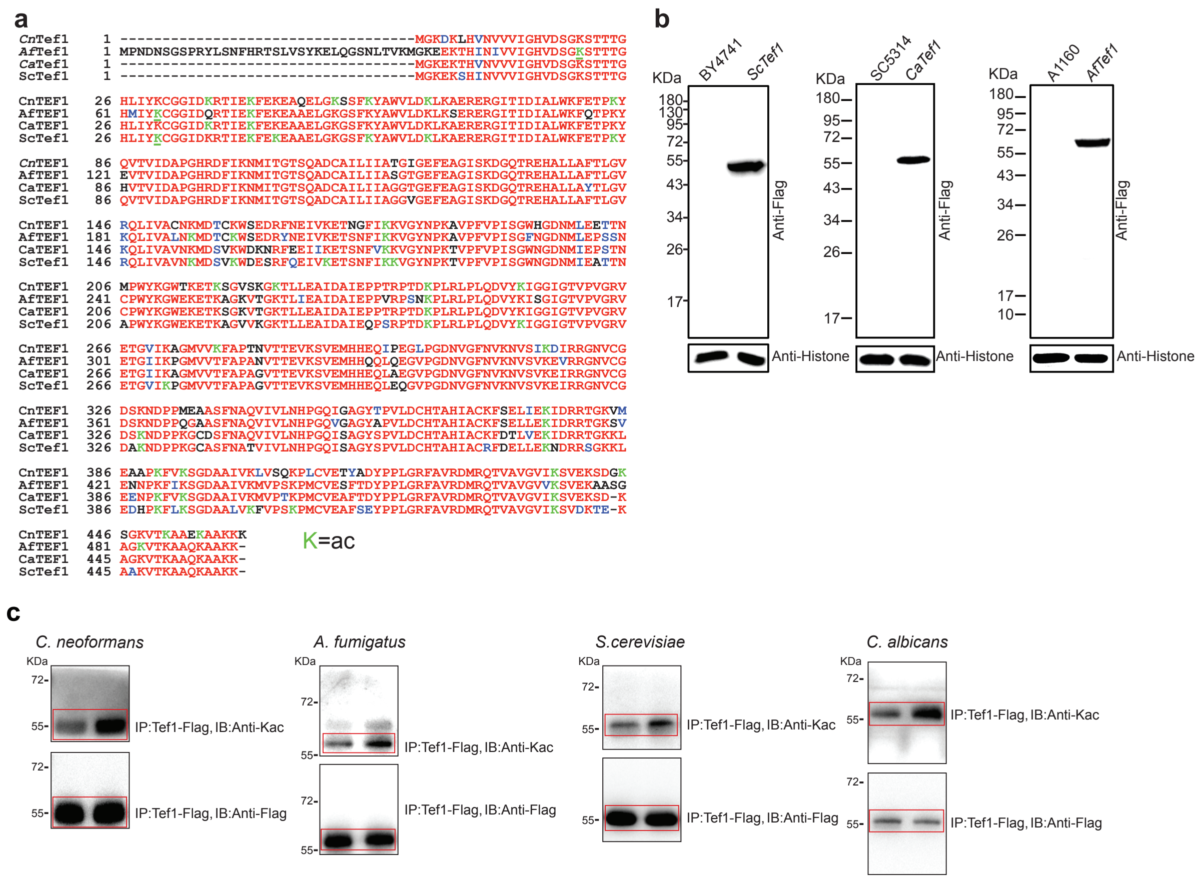

Supplementary Figure 2. Identification of Dac2 and Dac4 client proteins.

a. Multiple alignment of Tef1 proteins from *S. cerevisiae*, *C. neoformans*, *A. fumigatus*, and *C. albicans*. The multiple alignment was performed using Clustal Omega (https://www.ebi.ac.uk/Tools/msa/clustalo/).

b. Construction of *TEF1-FLAG* strains in *S. cerevisiae*, *C. albicans* and *A. fumigatus*. Immunoblotting was used to check for the expression of *TEF1* in the three fungal strains. Anti-Flag and anti-histone antibodies were used.

c. Full blots from Fig. 2b are presented. Indicated protein bands were cropped and shown in Fig. 2b.

a

Pearson Correlation		*dac2*Δ			*dac4*Δ			H99		
		repeat1	repeat2	repeat3	repeat1	repeat2	repeat3	repeat1	repeat2	repeat3
*dac2*Δ	repeat1	1.00	0.79	0.82	-0.51	-0.50	-0.51	-0.22	-0.08	-0.08
	repeat2	0.79	1.00	0.86	-0.43	-0.55	-0.51	-0.12	-0.21	-0.09
	repeat3	0.82	0.86	1.00	-0.44	-0.51	-0.50	-0.15	-0.13	-0.25
*dac4*Δ	repeat1	-0.51	-0.43	-0.44	1.00	0.88	0.90	-0.73	-0.68	-0.69
	repeat2	-0.50	-0.55	-0.51	0.88	1.00	0.93	-0.64	-0.70	-0.69
	repeat3	-0.51	-0.51	-0.50	0.90	0.93	1.00	-0.65	-0.68	-0.72
H99	repeat1	-0.22	-0.12	-0.15	-0.73	-0.64	-0.65	1.00	0.87	0.88
	repeat2	-0.08	-0.21	-0.13	-0.68	-0.70	-0.68	0.87	1.00	0.90
	repeat3	-0.08	-0.09	-0.25	-0.69	-0.69	-0.72	0.88	0.90	1.00

b

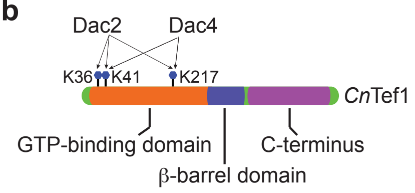

CNAG_06125	Ac (*dac2*Δ/WT)	Ac (*dac4*Δ/WT)
Tef1 K36	2.272	-
Tef1 K41	2.562	2.113
Tef1 K217	2.011	1.341

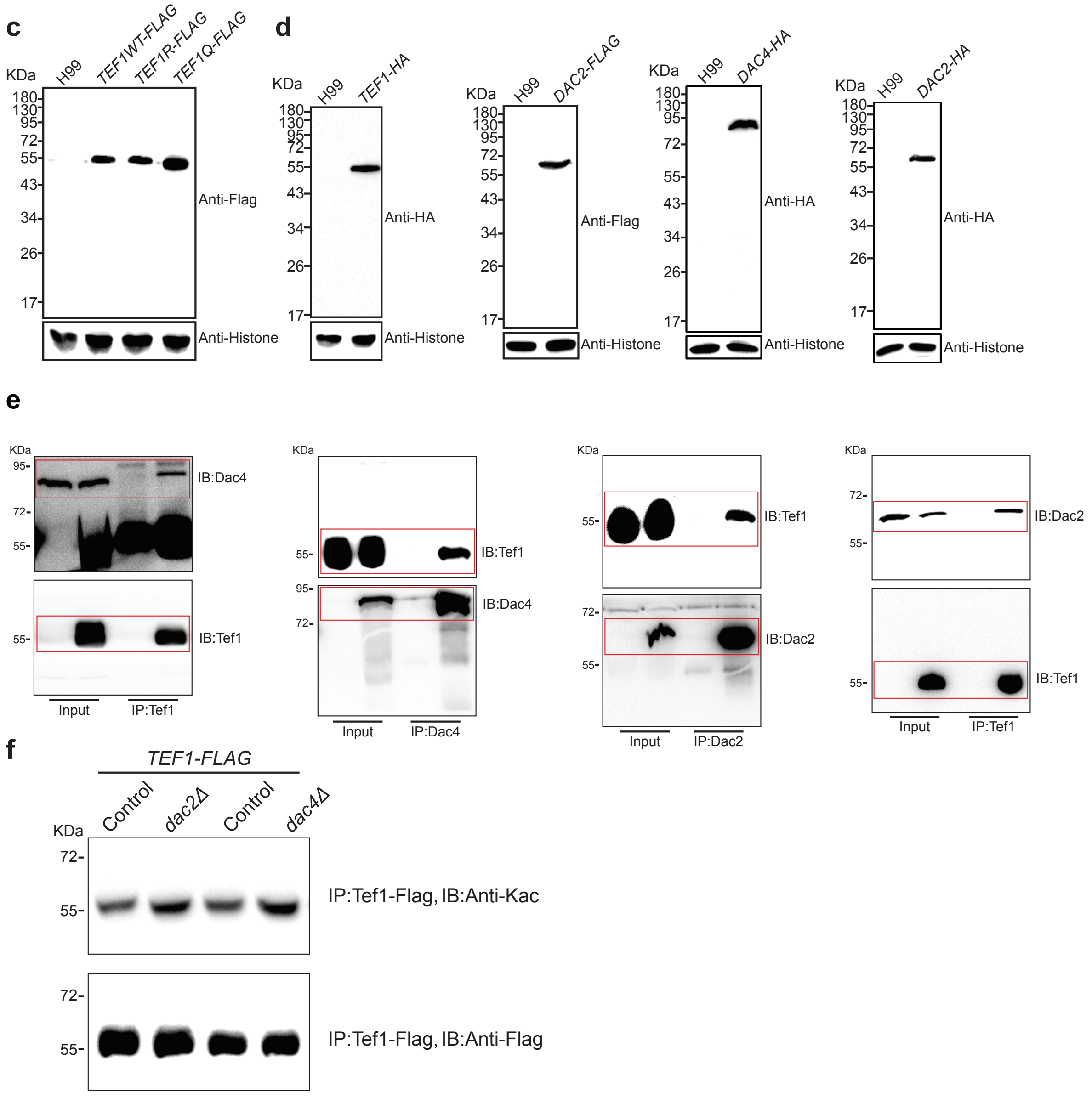

Supplementary Figure 3. Characterization of the acetylation of Tef1 in pathogenic fungi.

a. Reproducibility test of the acetylome analysis in *dac2Δ* and *dac4Δ*. The acetylome was performed in biological triplicates. Data correlations were calculated using Pearson correlation tests.

b. The acetylation sites of Tef1 (K36, K41, and K217) were upregulated in the *dac2Δ* strain. Sites K36 and K41 were upregulated in the *dac4Δ* strain.

c. Immunoblotting of *TEF1* mutants. An immunoblotting assay was performed to examine the expression of *TEF1* mutants. Protein samples of the wild-type, *TEF1WT-FLAG*, *TEF1R-FLAG* and *TEF1Q-FLAG* strains were isolated. Protein quantification was carried out using anti-Flag and anti-histone antibodies.

d. Immunoblotting of *TEF1* co-IP strains. Immunoblotting was performed using the wild-type, *TEF1-HA*, *DAC2-FLAG*, *DAC4-HA*, and *DAC2-HA* strains. Anti-HA, anti-Flag, and anti-histone antibodies were used for protein quantification.

e. Full blots from Figs. 3f and 3g are presented. Indicated protein bands are cropped and shown in Figs. 3f and 3g.

f. Analysis of Tef1 Kac levels in *C. neoformans dac2Δ* and *dac4Δ* strains. Protein samples from *dac2Δ* and *dac4Δ* strains were pulled down and quantified. Full blots are shown.

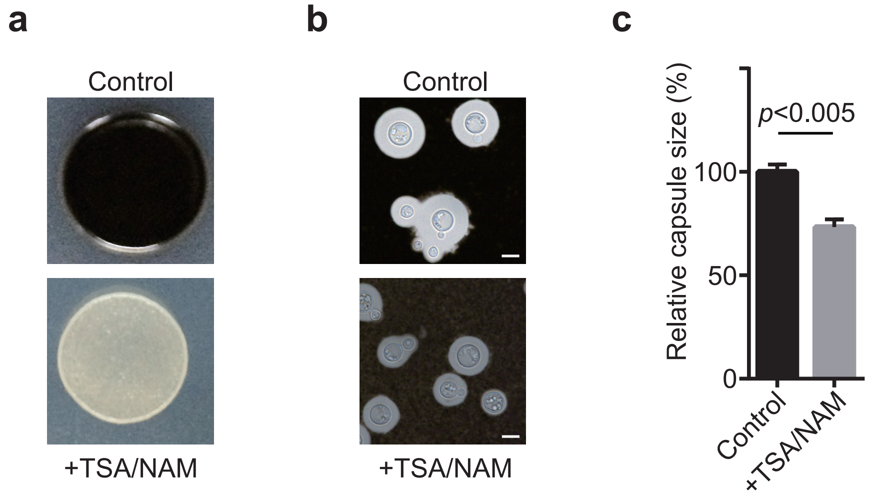

Supplementary Figure 4. TSA and NAM blocks the formation of melanin and capsule in *C. neoformans*.

a. Melanin formation. *C. neoformans* was spotted onto L-DOPA agar with or without 3 μM TSA and 20 mM NAM. Photographs were taken after 3 days of incubation.

b. Capsule production. Cells were incubated in Dulbeccos modified minimum essential medium (DMEM) with 10% FBS at 37°C and 5% CO_2.

c. Quantification of capsule. At least 100 cells were measured. Error bar indicates standard deviation (s.d.).

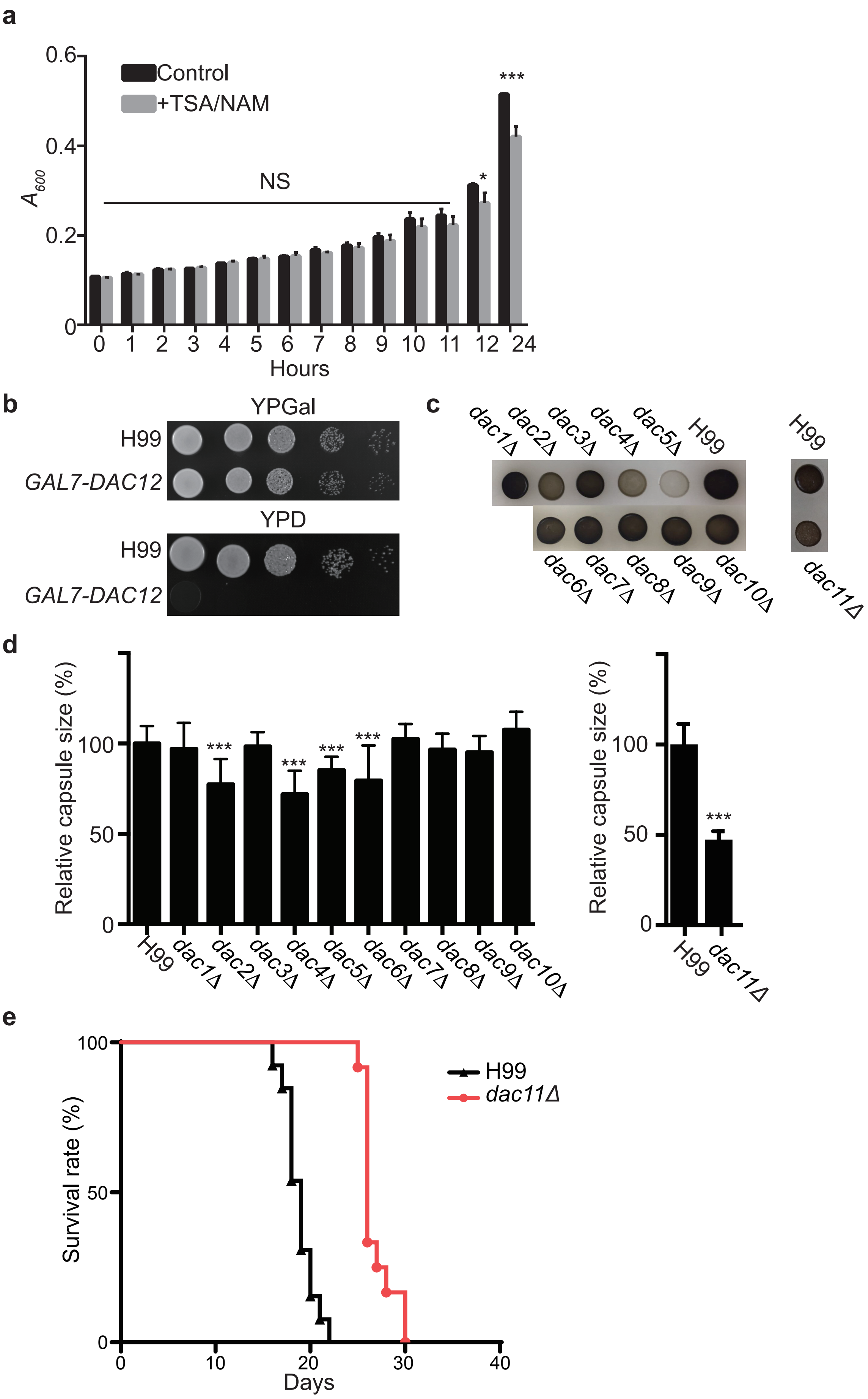
a
0.6
0.4
0.2
0
A600
Control
+TSA/NAM
NS
*
***
0 1 2 3 4 5 6 7 8 9 10 11 12 24
Hours
b
YPGal
H99
GAL7-DAC12
YPD
H99
GAL7-DAC12
c
dac1Δ dac2Δ dac3Δ dac4Δ dac5Δ H99
H99
dac6Δ dac7Δ dac8Δ dac9Δ dac10Δ
dac11Δ
d
Relative capsule size (%)
100
50
0
***
H99 dac1Δ dac2Δ dac3Δ dac4Δ dac5Δ dac6Δ dac7Δ dac8Δ dac9Δ dac10Δ
Relative capsule size (%)
H99 dac11Δ
e
Survival rate (%)
100
50
0
H99
dac11Δ
0 10 20 30 40
Days

Supplementary Figure 5. The characterization of *C. neoformans* deacetylase knockout strains.

a. Cell growth assay. *C. neoformans* cells were grown in YPD media with or without TSA/NAM, and cell densities were measured at the indicated time points at 600 nm. NS indicates non-significance.
b. Analysis of *GAL7-DAC12* strain. The *GAL-DAC12* cells were spotted onto YPGal (galactose) and YPD media. Cells were grown at 30°C for three days.
c. Melanin formation of deacetylase gene knockout strains. Genes encoding *C. neoformans* deacetylases were knocked out. The knockout strains were spotted onto L-DOPA agar plates and incubated at 37°C for three days.
d. Capsule production of deacetylase gene knockout strains. The knockout strains were incubated in DMEM supplemented with 10% FBS. The cultures were incubated 3 days, and cells were stained with India ink. Capsule thicknesses were measured and quantified. At least 100 cells were measured for each strain.
e. Animal survival curve of *dac11Δ* strain. Mice (n=10) were infected with *C. neoformans* H99 or *dac11Δ* strain. Mice survival rates were recorded. A Kaplan-Meier survival chart was plotted.

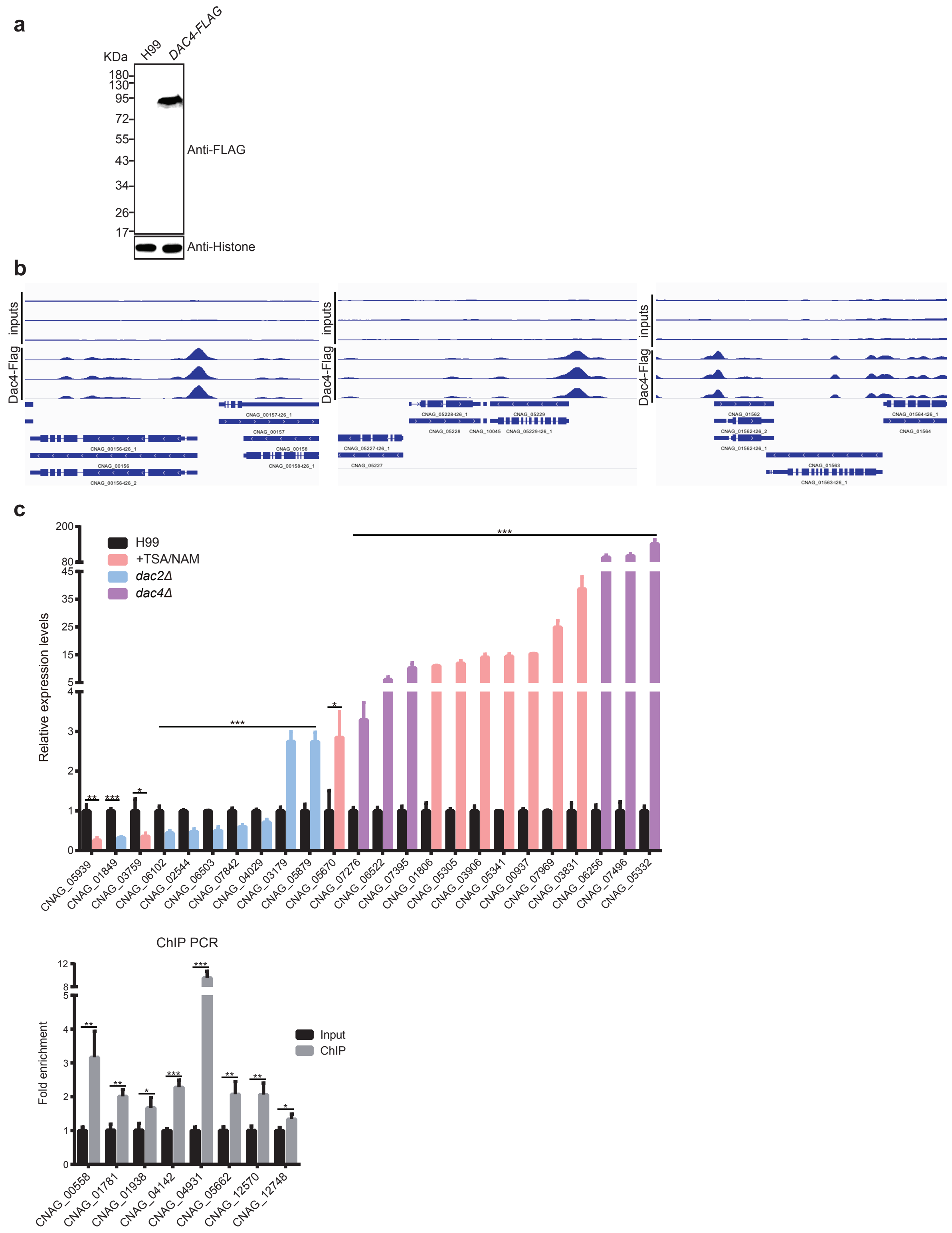

Supplementary Figure 6. Construction of *dac2Δ* and *dac4Δ* regulation networks.

a. The generation of the strain expressing Dac4-Flag. The *DAC4-FLAG* integration cassette was transformed into *C. neoformans*. The protein expression of Dac4-Flag was confirmed using immunoblotting. A single band was detected using an anti-Flag

antibody, while no signal was detected for the wild-type strain. The constructed strain was used for the ChIP-seq analysis.

b. ChIP-seq analysis of Dac4-Flag. ChIP-seq was performed in the *DAC4-FLAG* strain. Three biological replicates were used. The data was visualized using the Integrative Genome Viewer (IGV v2.4) [41]. Representative targets were shown, where the bindings of Dac4-Flag were enriched for CNAG_00157, CNAG_05229 and CNAG_01563 (*DAC4*).

c. Confirmation of RNA-seq and ChIP-seq data. Real-time PCR analyses were performed to verify RNA-seq and ChIP-seq results. RNA samples were isolated from cells treated with TSA/NAM, H99, *dac2Δ*, and *dac4Δ* cells. ChIP assays were performed using the *DAC4-FLAG* strain. Validation targets were randomly selected, and at least three biological replicates were performed for each. Alterations in gene expression or enrichment were calculated by comparing to the reference strain or input samples.

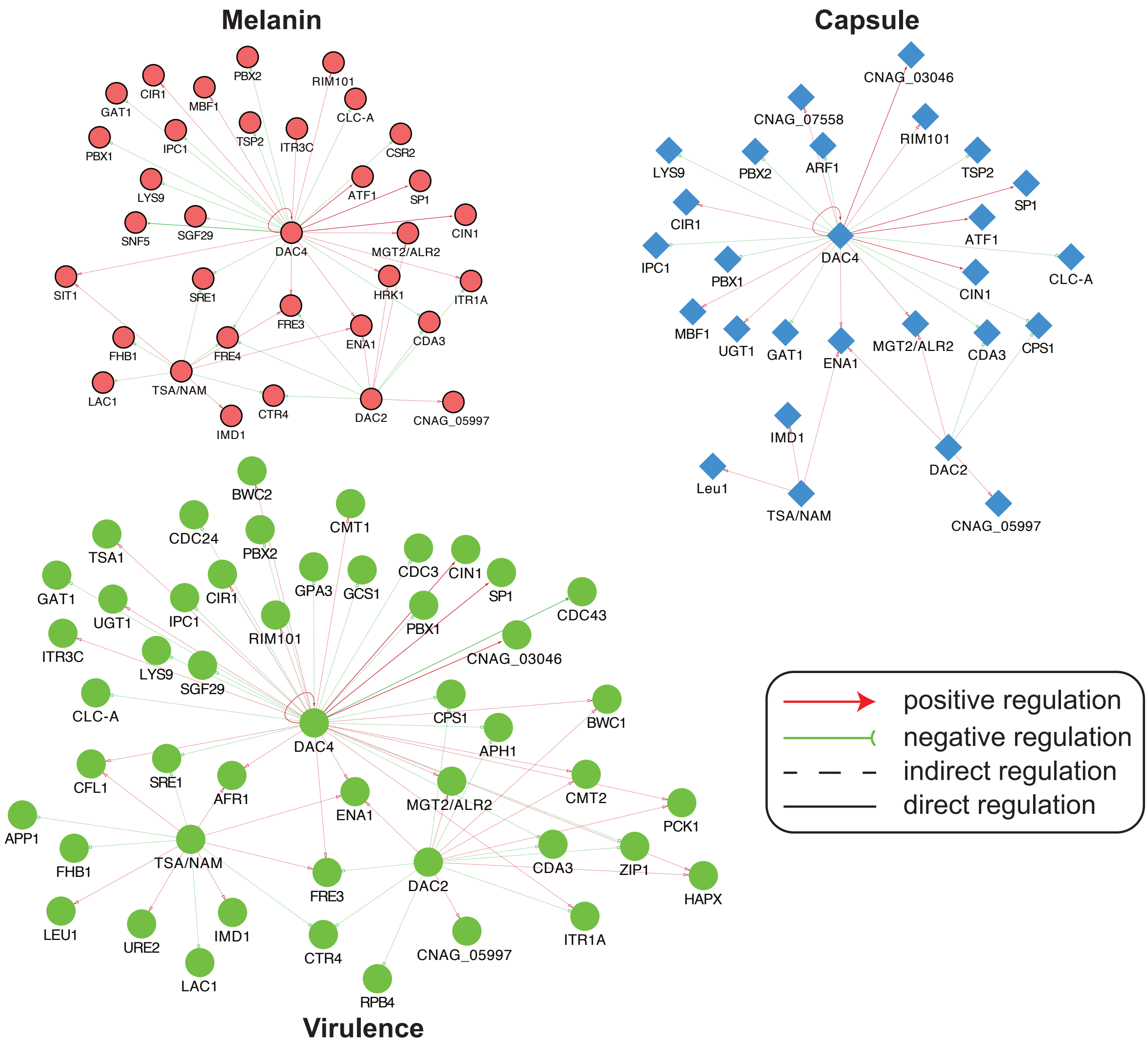

Supplementary Figure 7. Regulatory networks of Dac2 and Dac4.

The regulatory networks of known modulators of melanin, capsule and virulence are illustrated.

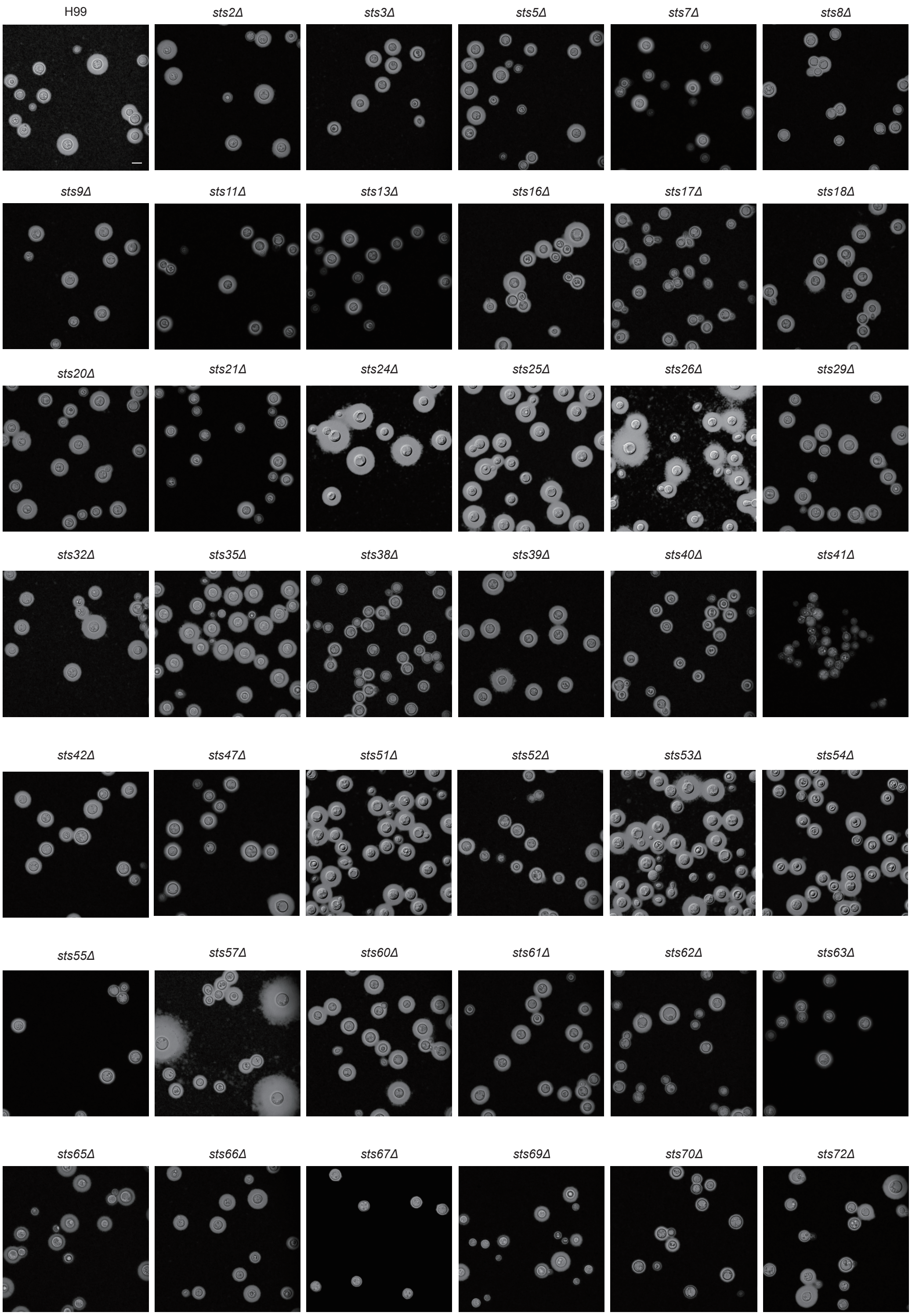

Supplementary Figure 8. Capsule formation of *C. neoformans* knockout mutants.

The knockout strains were incubated in DMEM supplemented with 10% FBS. The cultures were incubated 3 days, and cells were stained with India ink. Capsule structure was visualized and photographed.

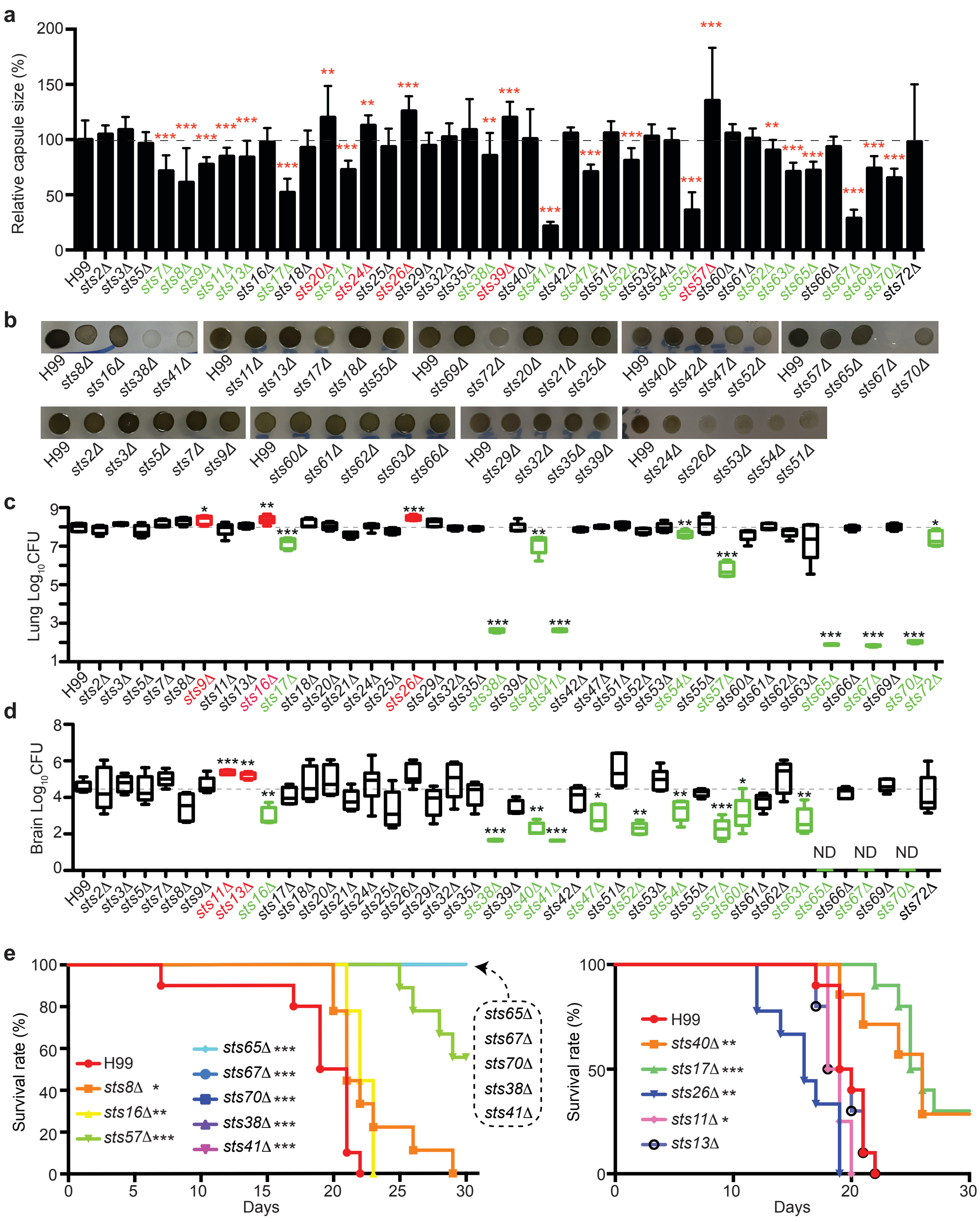

Supplementary Figure 9. Screen for novel virulence factors in *C. neoformans*.

a. Capsule production in *C. neoformans* mutants. Capsule thicknesses were measured and quantified. At least 100 cells were measured for each strain. Error bar indicates s.d.

b. Melanin formation in *C. neoformans* mutants.

c. Lung fungal burden in mutants. Mice were infected with *C. neoformans* mutants. CFUs were assayed and plotted using box plot. Red boxes indicate inductions in CFUs, and green boxes represent attenuations in CFUs.

d. Brain fungal burden in mutants.

e. Animal survival assays. Kaplan-Meier survival charts were plotted. Mice (n=10) were infected with *C. neoformans* H99 or indicated mutant strains. Mice survival rates were recorded. A Kaplan-Meier survival chart was plotted.

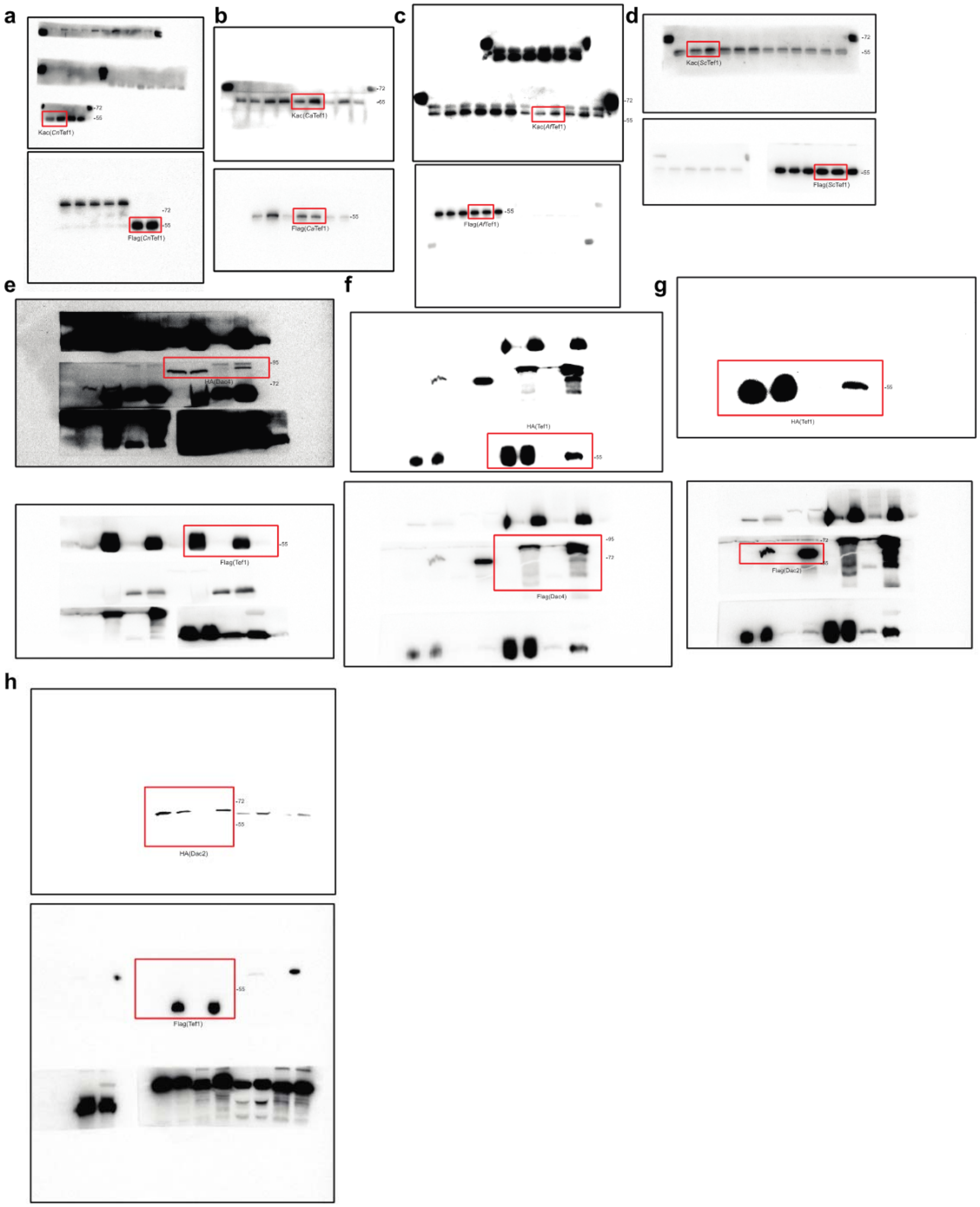

Supplementary Figure 10: Immunoblotting raw data.

a) Raw data for *C. neoformans* Tef1 Kac assay in Figure 2b. **b)** Raw data for *C. albicans* Tef1 Kac assay in Figure 2b. **c)** Raw data for *A. fumigatus* Tef1 Kac assay in Figure 2b. **d)** Raw data for *S. cerevisiae* Tef1 Kac assay in Figure 2b. **e)** Raw data for Figure 3f (top panel). **f)** Raw data for Figure 3f (bottom panel). **g)** Raw data for Figure 3g (top panel). **h)** Raw data for Figure 3g (bottom panel).
