# Supplementary material for: Fungal acetylome comparative analysis identifies an essential role of acetylation in human fungal pathogen virulence
Source: Commun Biol. 2019 May 1;2:154. doi: 10.1038/s42003-019-0419-1 (PMC6494858; doi:10.1038/s42003-019-0419-1)
Supplement: Supplementary file 10 — Description of Supplementary Data [file 42003_2019_419_MOESM10_ESM.pdf]

Description of Additional Supplementary Files

File Name: Supplementary Data 1
Description: **Acetylome analyses in *C. neoformans, A. fumigatus* and *C. albicans*[a,b,c,d,e].**

[a]Acetylome analysis in *C. neoformans*. The acetylome data of three biological replicates of *dac4Δ*, three biological replicates of *dac2Δ*, and five biological replicates of H99 were pooled.

[b]Acetylome analysis in *A. fumigatus*. The acetylome data of three biological replicates of Af293 were pooled.

[c]Acetylome analysis in *C. albicans*. The acetylome data of three biological replicates of SC5314 were pooled.

[d]Comparative acetylome analysis in *dac4Δ* and H99. The acetylome data of H99 and *dac2Δ* cells were normalized to proteome data, and the ratios of Kac levels were then calculated. Three biological replicates were performed.

[e]Comparative acetylome analysis in *dac2Δ* and H99. The acetylome data of H99 and *dac2Δ* cells were normalized to proteome data, and the ratios of Kac levels were then calculated. Three biological replicates were performed.

File Name: Supplementary Data 2
Description: **Kac orthologs and sites analysis in *C. neoformans, A. fumigatus, C. albicans* and *S. cerevisiae*[a,b].**

[a]Kac ortholog analysis in fungi. Orthologous relationship of *C. neoformans*, *A. fumigatus*, *C. albicans* and *S. cerevisiae* was downloaded from FungalDB (http://fungidb.org/fungidb/). Orthologs identified in the acetylome results are shown in red.

[b]Kac site analysis in fungi. Ortholog information of *C. neoformans*, *A. fumigatus*, *C. albicans* and *S. cerevisiae* was downloaded from FungalDB (http://fungidb.org/fungidb/) and proteins from each ortholog group were aligned using Clustal Omega (https://www.ebi.ac.uk/Tools/msa/clustalo/). The conservation of lysine residues within an ortholog group were then extracted from the alignment files. The conservation of acetylation of lysine residues were then analyzed based on our fungal acetylome data. Non-conserved lysine residues are shown as AA-ID (residue-ortholog ID). Conserved lysine acetylation residues are

shown as Kac-ortholog ID. Conserved lysine residues that were not identified in the acetylome are indicated as nK- ortholog ID.

File Name: Supplementary Data 3
Description: **Literature information of characterized pathogenicity factors in *C. neoformans, C. albicans* and *A. fumigatus* identified in the transcriptome and acetylome.**

File Name: Supplementary Data 4
Description: **Transcriptome analysis in *C. neoformans* knock-outs or wild-type cells treated with deacetylase inhibitors[a,b,c,d,e,f,g].**
[a]*C. neoformans* H99 cells were grown in YPD medium supplemented with (or without) TSA and NAM. RNA samples were isolated and subjected to transcriptome analysis. Genes with differential expressions greater than 1.5 or less than 0.667, and padj<0.05 were considered to have significantly changed.
[b]*C. neoformans* H99 and *dac2Δ* cells were grown in YPD medium. RNA samples were isolated and subjected to transcriptome analysis. Genes with differential expressions greater than 1.5 or less than 0.667, and padj<0.05 were considered to have significantly changed.
[c]*C. neoformans* H99 and *dac4Δ* cells were grown in YPD medium. RNA samples were isolated and subjected to transcriptome analysis. Genes with differential expressions greater than 1.5 or less than 0.667, and padj<0.05 were considered to have significantly changed.
[d]Comparative transcriptome analysis using *dac2Δ* and *dac4Δ* transcriptome data.
[e]Comparative transcriptome analysis using *dac4Δ* and TSA/NAM transcriptome data.
[f]Comparative transcriptome analysis using *dac2Δ* and TSA/NAM transcriptome data.
[g]Comparative transcriptome analysis using *dac2Δ*, *dac4Δ* and TSA/NAM transcriptome data.

File Name: Supplementary Data 5
Description: **ChIP-seq analysis in the *DAC4-FLAG* strain.**

File Name: Supplementary Data 6
Description: **Kac motif analysis in *C. neoformans, A. fumigatus, C. albicans,* and *S. cerevisiaea*.**
Kac motif analyses in fungi. 20 amino acids flanking the Kac site were examined for enrichment. Fisher tests were used to calculate the significance. -Log10 p-values are indicated. Red indicates

significantly enriched amino acids; green or white squares suggest low possibility or the unbiased occurrence of amino acids, respectively.

File Name: Supplementary Data 7
Description: **Strains used in this study.**

File Name: Supplementary Data 8
Description: **Strains used in this study.**
