# Supplementary material for: Fungal acetylome comparative analysis identifies an essential role of acetylation in human fungal pathogen virulence
Source: Commun Biol. 2019 May 1;2:154. doi: 10.1038/s42003-019-0419-1 (PMC6494858; doi:10.1038/s42003-019-0419-1)
Supplement: Supplementary file 11 — Reporting Summary [file 42003_2019_419_MOESM11_ESM.pdf]

nature research

Corresponding author(s): Chen Ding

Last updated by author(s): Mar 26, 2019

Reporting Summary

Nature Research wishes to improve the reproducibility of the work that we publish. This form provides structure for consistency and transparency in reporting. For further information on Nature Research policies, see Authors & Referees and the Editorial Policy Checklist.

Statistics

For all statistical analyses, confirm that the following items are present in the figure legend, table legend, main text, or Methods section.

n/a	Confirmed	
☐	☒	The exact sample size (*n*) for each experimental group/condition, given as a discrete number and unit of measurement
☐	☒	A statement on whether measurements were taken from distinct samples or whether the same sample was measured repeatedly
☐	☒	The statistical test(s) used AND whether they are one- or two-sided<br>*Only common tests should be described solely by name; describe more complex techniques in the Methods section.*
☐	☒	A description of all covariates tested
☐	☒	A description of any assumptions or corrections, such as tests of normality and adjustment for multiple comparisons
☐	☒	A full description of the statistical parameters including central tendency (e.g. means) or other basic estimates (e.g. regression coefficient) AND variation (e.g. standard deviation) or associated estimates of uncertainty (e.g. confidence intervals)
☐	☒	For null hypothesis testing, the test statistic (e.g. *F*, *t*, *r*) with confidence intervals, effect sizes, degrees of freedom and *P* value noted<br>*Give P values as exact values whenever suitable.*
☒	☐	For Bayesian analysis, information on the choice of priors and Markov chain Monte Carlo settings
☒	☐	For hierarchical and complex designs, identification of the appropriate level for tests and full reporting of outcomes
☐	☒	Estimates of effect sizes (e.g. Cohen's *d*, Pearson's *r*), indicating how they were calculated

Our web collection on statistics for biologists contains articles on many of the points above.

Software and code

Policy information about availability of computer code

Data collection	Differentially expressed genes and acetylated proteins were searched for orthologs using FungiDB (http://fungidb.org/fungidb/).<br>The ChIP-seq raw reads were mapped to the C. neoformans H99 genome (downloaded from http://fungidb.org/fungidb/) using the Bowtie 2suite (version 4.1.2) (Langmead and Salzberg, 2012).<br>Kac motif analysis:The model of sequences constituting with amino acids in specific positions of modify-21-mers (10 amino acids upstream and downstream of the Kac site) in all protein sequences were downloaded at www.uniprot.org and analyzed.
Data analysis	The ChIP-seq library was constructed using the MicroPlex Library Preparation Kit v2 (Diagenode) according to the manufacturer's instructions. The ChIP DNA libraries were sequenced using an Illumina HiSeq 2500 Platform. The SAM files were then sorted using SAMtools (version 1.3.1) (Li et al., 2009). The ChIP-seq peaks were analyzed using the MACS suite (Model-based analysis of ChIP- seq, version 1.4.2) with a p-value cutoff of 10-8 (Zhang et al., 2008). The ChIP-seq signal was visualized using IGV (Integrative Genomic Viewer, version 2.3.98) (Robinson et al., 2011).<br>The conserved Kac sites were determined from ortholog multiple alignment using Clustal Omega (https://www.ebi.ac.uk/Tools/msa/clustalo/). Aligned K residues that are detected in our acetylome analysis are considered as conserved Kac sites. The regulatory networks of differentially expressed genes or acetylated proteins were constructed using Cytoscape (version 3.6.0) (Shannon et al., 2003).<br>Information regarding the functions of identified genes or proteins in the regulation of fungal virulence were obtained from published literature (https://www.ncbi.nlm.nih.gov/pubmed).

For manuscripts utilizing custom algorithms or software that are central to the research but not yet described in published literature, software must be made available to editors/reviewers. We strongly encourage code deposition in a community repository (e.g. GitHub). See the Nature Research guidelines for submitting code & software for further information.

Data

Policy information about availability of data

All manuscripts must include a data availability statement. This statement should provide the following information, where applicable:

- Accession codes, unique identifiers, or web links for publicly available datasets
- A list of figures that have associated raw data
- A description of any restrictions on data availability

- Accession codes, unique identifiers, or web links for publicly available datasets
-The raw mass spectrometry data of acetylome and proteome data have been deposited to the ProteomeXchange (https://www.ebi.ac.uk/pride) with identifier PXD010354. The transcriptome (RNA-seq) and ChIP-seq data are deposited in NCBI's Gene Expression Omnibus (GEO) (https://www.ncbi.nlm.nih.gov/geo/) and can be accessed through GEO Series accession ID GEO: GSE116040.
- A list of figures that have associated raw data
-Figure 1, Figure 2a, Figure 3c, Figure 4, Figure 5a, Figure 5b, Figure 6, Figure 7, Supplemental figure 1, Supplemental figure 2a, Supplemental figure 3a, Supplemental figure 3b, Supplemental figure 6b, Supplemental figure 6c, Supplemental figure 7
- A description of any restrictions on data availability
-No restrictions on data availability

Field-specific reporting

Please select the one below that is the best fit for your research. If you are not sure, read the appropriate sections before making your selection.

☒ Life sciences ☐ Behavioural & social sciences ☐ Ecological, evolutionary & environmental sciences

For a reference copy of the document with all sections, see nature.com/documents/nr-reporting-summary-flat.pdf

Life sciences study design

All studies must disclose on these points even when the disclosure is negative.

Sample size	The mass errors of identified Kac peptides in C. neoformans, C. albicans and A. fumigatus. C. neoformans acetylome data contains the acetylome data that includes three biological replicates from dac2Δ, three biological replicates from dac4Δ and three biological replicates from the H99 strain, and also includes two additional replicates from the H99 strains, grown at 30°C or 37°C.<br>The RNA-seq includes three biological replicates from dac2Δ, three biological replicates from dac4Δ and three biological replicates from the H99 strain.<br>ChIP-seq was performed in the DAC4-FLAG strain. Three biological replicates were used.<br>C57BL/6 mice (n=7) were infected with C. neoformans for Fungal Burden Assay and C57BL/6 mice (n=10) for survival.<br>Capsule thicknesses were measured and quantified. At least 100 cells were measured for each strain.
Data exclusions	The sequencing data was normalized by corresponding softwares or programs, and no experimental data was excluded.
Replication	Three biological replicates were done for RNA-seq and ChIP-seq.
Randomization	All animal and sequencing experiments are allocated randomly.
Blinding	The data collections or analyses are blinded for all investigators.

Reporting for specific materials, systems and methods

We require information from authors about some types of materials, experimental systems and methods used in many studies. Here, indicate whether each material, system or method listed is relevant to your study. If you are not sure if a list item applies to your research, read the appropriate section before selecting a response.

Materials & experimental systems

n/a	Involved in the study
☐	☒ Antibodies
☒	☐ Eukaryotic cell lines
☒	☐ Palaeontology
☐	☒ Animals and other organisms
☒	☐ Human research participants
☒	☐ Clinical data

Methods

n/a	Involved in the study
☐	☒ ChIP-seq
☒	☐ Flow cytometry
☒	☐ MRI-based neuroimaging

Antibodies

Antibodies used	Mouse anti-acetyllysine primary antibody (clone Kac-01), PTM Bio, Cat# PTM-101;<br>Rabbit anti-acetyllysine primary antibody, PTM Bio, Cat# PTM-105;<br>Rabbit anti-HA primary antibody, Abcam, Cat# AB9110;<br>Rabbit anti-FLAG primary antibody, Abcam, Cat# AB1162;<br>Rabbit anti-Histone H3 (D1H2) primary antibody, Cell Signaling Technology, Cat# 4499;<br>Goat anti-Mouse IgG (H+L) Secondary Antibody, Invitrogen, Cat# 31430;<br>Goat anti-Rabbit IgG (H+L) Secondary Antibody Invitrogen Cat# 31460.
Validation	See Figure 2b, Figure 3f, Figure 3g, Supplemental figure 2b, Supplemental figure 2c, Supplemental figure 3c, Supplemental figure 3d, Supplemental figure 3e, Supplemental figure 3f, Supplemental figure 6a

Animals and other organisms

Policy information about studies involving animals; ARRIVE guidelines recommended for reporting animal research

Laboratory animals	Six- to eightweek-old female C57BL/6 mice per fungal strain were used for survival and fungal burden analysis. Infected mice were sacrificed on post-infection day 14 using carbon dioxide.
Wild animals	The study did not involve wild animals
Field-collected samples	The study did not involve field-collected animals
Ethics oversight	All animal experiments were carried out under the review and approval of the Research Ethics Committees at the College of Life and Health Sciences of Northeastern University.

Note that full information on the approval of the study protocol must also be provided in the manuscript.

ChIP-seq

Data deposition

☒ Confirm that both raw and final processed data have been deposited in a public database such as GEO.

☒ Confirm that you have deposited or provided access to graph files (e.g. BED files) for the called peaks.

Data access links<br>*May remain private before publication.*	To review GEO accession GSE116040:<br>Go to https://www.ncbi.nlm.nih.gov/geo/query/acc.cgi?acc=GSE116040<br>Enter token uhuhmemkrhozrqd into the box
Files in database submission	GSM3207837 ChIP.rep1<br>GSM3207838 ChIP.rep2<br>GSM3207839 ChIP.rep3<br>GSM3207840 ChIP-Input.rep1<br>GSM3207841 ChIP-Input.rep2<br>GSM3207842 ChIP-Input.rep3
Genome browser session<br>(e.g. UCSC)	http://fungidb.org/fungidb/

Methodology

Replicates	Three replicates: Chip.rep1, Chip.rep2, Chip.rep3, Chip-Input.rep1, Chip-Input.rep2, Chip-Input.rep3
Sequencing depth	ChIP-seq rep1:<br>4571226 reads; 96.67% overall alignment rate<br>ChIP-seq rep2:<br>5047946 reads; 96.87% overall alignment rate<br>ChIP-seq rep3:<br>4171159 reads; 96.74% overall alignment rate<br>Input rep1:<br>3312003 reads; 95.89% overall alignment rate<br>Input rep2:<br>4337450 reads; 95.76% overall alignment rate<br>Input rep3:<br>3885412 reads; 95.59% overall alignment rate<br>Read length: 150 bp<br>Read type: Paired-end
Antibodies	Rabbit anti-FLAG primary antibody， Abcam, Cat# AB1162

Peak calling parameters	Peaks were called using MACS 1.4.2 suite with default parameters and a p-value cut off of 10-8 was used (macs14 -t ChIP.sam -c Input.sam -n name –p 10-8 -w).
Data quality	At the p-value threshold of enrichment of 10-8, all peaks enriched at FDR <5% and over 70% peaks showed > 5-fold enrichment.<br>To ensure the data quality, we overlapped the peaks called from all three-independent experiment.
Software	The ChIP DNA libraries were sequenced using an Illumina HiSeq 2500 Platform. Raw reads were mapped to the C. neoformans H99 genome (downloaded from http://fungidb.org/fungidb/) using the Bowtie 2 suite (version 4.1.2) (Langmead and Salzberg, 2012). The SAM files were then sorted using SAMtools (version 1.3.1) (Li et al., 2009). The ChIPseq signal was visualized using IGV (Integrative Genomic Viewer, version 2.3.98) (Robinson et al., 2011).
